# Injectable and Conductive Polyurethane Gel with Load-Responsive Antibiosis for Sustained Root Canal Disinfection

**DOI:** 10.3390/gels11050346

**Published:** 2025-05-07

**Authors:** Bo Mu, Xiaoyu Lei, Yinglong Zhang, Jingzheng Zhang, Qingda Du, Yuping Li, Dongyu Huang, Li Wang, Jidong Li, Yubao Li, Yi Zuo

**Affiliations:** 1 Research Center for Nano Biomaterials, Analytical & Testing Center, Sichuan University, Chengdu 610064, China; mubo1205@163.com (B.M.); zehn0906@163.com (X.L.); zhangyinglong1996@163.com (Y.Z.); scuzjz@163.com (J.Z.); duqingda0309@163.com (Q.D.); liyuping09115@163.com (Y.L.); h_dongy@163.com (D.H.); nic1979@scu.edu.cn (J.L.); nic7504@scu.edu.cn (Y.L.); 2 School of Big Health and Intelligent Engineering, Chengdu Medical College, Chengdu 610500, China; liwang@cmc.edu.cn

**Keywords:** root canal therapy, piezoelectric antibacterial gel, mechano-electric coupling, biofilm eradication, sustained antibacterial

## Abstract

To address the limitations of conventional antibacterial therapies, we developed an injectable, conductive polyurethane-based composite gel system for sustained root canal disinfection. This gel incorporates piezoelectric nanoparticles (n-BaTiO_3_) and conductive segments (aniline trimer, AT) within a polyurethane matrix, which synergistically interact with a static antimicrobial agent (n-ZnO) to achieve dynamic, mechano-responsive antibacterial activity. Under cyclic compression (simulating mastication), the piezoelectric gels exhibited enhanced electroactivity via the mechano-electric coupling effect, generating 2-fold higher voltage and a 1.8–1.9× increase in current compared to non-piezoelectric controls. The dynamic electroactivity of the gels enabled superior long-term performance, achieving 92–97% biofilm eradication, significantly higher than the static n-ZnO-only gel (88%). XPS and UV-vis spectroscopy analyses confirmed mechano-electrochemically amplified reactive oxygen species (ROS) generation, which contributed to improved biofilm disruption. The ISO-compliant gel provides durable, load-responsive disinfection while maintaining good biocompatibility, offering a promising solution to prevent post-treatment reinfection.

## 1. Introduction

As the second largest microbial ecosystem in humans, the oral cavity harbors over 700 bacterial species and approximately 100 fungal species. Recent metagenomic studies have revealed that 296 species-level microbial populations typically coexist within this habitat [1,2]. Generally, the overgrowth of pathogenic bacteria contributes to various oral diseases, including dental caries, gingivitis, periodontitis, candidiasis, and diverse infections [3,4,5]. Disinfection is a clinical intervention aimed at controlling bacterial proliferation. However, excessive antibiotic use may disrupt the balance of the oral microbiota and foster antibiotic resistance, potentially contributing to severe systemic diseases [6,7,8]. Root canal therapy becomes essential when tooth integrity is compromised and pulp removal becomes necessary [9]. Root canal filling often represents the last attempt to salvage a tooth affected by irreversible inflammation or necrosis due to caries, structural defects (e.g., cracks or chips), or traumatic dental injuries [10]. The failure of root canal therapy is frequently attributed to bacterial reinfection following the decline in antibacterial activity over time, or more critically, inadequate obturation caused by the shrinkage of filling materials [9]. Furthermore, the oral environment is harsh and intricate, providing an ideal breeding ground for bacteria to form biofilms [11]. Within these dense biofilms, bacteria can evade the effects of antibiotics [12], posing a significant challenge to achieving sustained antibacterial efficacy in the treatment of infected root canals.

Naturally, the tooth serves as a direct tool for performing the chewing function. During chewing, the contraction of masticatory muscles induces occlusion between the upper and lower teeth [13,14]. According to reports, the theoretical maximum bite force between opposing teeth can reach 200–500 kg [15]. Chewing force enhances the efficiency of food digestion and facilitates the absorption and utilization of nutrients. During mastication, occlusal forces generate localized stress concentrations at specific anatomical sites, such as tooth interfaces and periodontal tissues [15]. This stress is transmitted to the jaw through the teeth, aiding in maintaining the alignment and position of the teeth and jawbone. Furthermore, the stress stimulates periodontal tissues, promoting the differentiation of periodontal cells and eliminating inflammatory factors induced by interleukin-1β, thus exerting an anti-inflammatory effect [16,17,18]. Stress stimulation supports the blood circulation and metabolism of periodontal tissues, thereby maintaining their health and ensuring dental stability [17]. Consequently, the development of a new type of filling cement has been proposed, leveraging the mechanical environment of the oral cavity to achieve sustained antibacterial properties, which could significantly reduce the proliferation of harmful bacteria in root canal therapy.

The efficacy of endodontic usage testing is required to last longer than 28 ± 3 days according to ISO 7405:2018 [19]. However, the antibacterial effects of some root canal filling materials cannot persist beyond one week due to the limited release of antibacterial substances or temporary alterations in pH. Examples include Epiphany SE root canal filling, Pulp Canal Sealer EWT, and EndoSequence BC root canal filling [10]. Incorporating inorganic antibacterial components into root canal filling materials has emerged as an effective strategy to enhance their antibacterial properties, such as silver and copper salts, as well as zinc and titanium oxides [20,21,22,23]. Nevertheless, the action mechanisms of these inorganic antibacterial agents—whether through contact inhibition or diffusional bacteriostasis—are highly dependent on environmental conditions. Consequently, their sustained release is often restricted in the dry and dark root canal environment, which is essential for hard curing [24].

Few root canal filling materials have been considered, despite the significant compressive stress generated daily in the oral environment. It has been reported that piezoelectric membranes can continuously generate electrical charges under oral pressure, contributing to bacterial elimination and wound healing [25]. This class of materials exhibits clinical utility across various biomedical domains, facilitating advancements in osseous regeneration, controlled therapeutic release, and engineered tissue reconstruction [26]. Dentistry could benefit from the piezoelectric effect, given an average of 500,000 chewing cycles per year [27]. Compared to lead zirconate titanate, barium titanate nanoparticles (n-BaTiO_3_) are a simpler chemical compound with excellent piezoelectric properties and good biocompatibility, making them suitable for the complex oral environment [28]. Incorporating piezoelectric BaTiO_3_ nanoparticles into a Bis-GMA/TEGDMA (1:1) dental composite matrix demonstrates piezoresponsive behavior under mechanical loading. This results in significantly reduced bacterial growth and pathogenic biofilm formation through the production of reactive oxygen species (ROS). The composite was specifically developed for restorative dental applications, such as tooth surface repairs, to prevent secondary caries at the bonded interface [29,30]. However, the dipole rearrangement-generated charges are confined to the surface of inorganic particles, which can be easily masked by insulating polymers during synthesis, thereby weakening the electric signals [31]. Consequently, the piezoelectric potential is limited in its efficacy for electronic transmission on the outer surface of filling materials.

**Figure 1:**
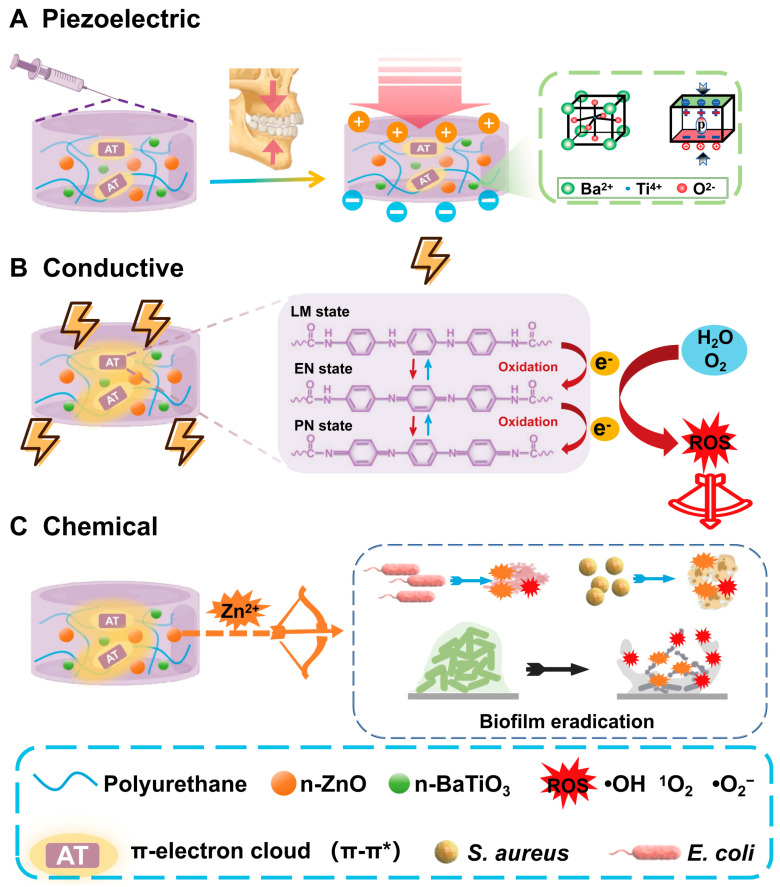
**Schematic illustration of the injectable and conductive polyurethane gel for root canal therapy:** (**A**) Piezoelectric nanoparticles (n-BaTiO_3_) synergistically cooperated (**B**) conductive AT segments in polyurethane chains with (**C**) chemical antimicrobial agent (n-ZnO) to develop a series of mechano-powered antibacterial gels with dynamically sustained bacteriostasis as root canal filling.

In our prior research, we fabricated a series of conductive polyurethane (CPU) fibrous membranes incorporating aniline trimer (AT), inspired by the periosteum, to promote bone regeneration [32]. The fibrous membrane shows good biocompatibility after subcutaneous implantation. However, due to the formation of a crosslinked network within the matrix, the conductive polymer cannot serve as a self-curing root canal filling material because it results in an organogel with inadequate injectability [33]. In this study, a series of injectable conductive polyurethane gels incorporating piezoelectric n-BaTiO_3_ and other functional components were developed as a sustained bacteriostasis strategy for root canal therapy (Figure 1). These injectable conductive gels exhibit varying electroactivity under cyclic compressive loading conditions as well as in the absence of mechanical stress. Furthermore, the antibacterial efficacy of these electrically activated gels was specifically evaluated against bacteria and biofilms under different cyclic loading forces. Inspired by their high piezoelectric activity under pressure, in vitro studies revealed that the novel self-powered gels based on conductive polyurethane significantly enhanced antibacterial performance compare to chemical bactericide. The dynamic strategy is expected to achieve a sustained antibacterial effect in the oral chewing environment through the mechano-electric integrated effect.

## 2. Results and Discussion

### 2.1. Synthesis of AT and CPU-Based Prepolymers

**Figure 2:**
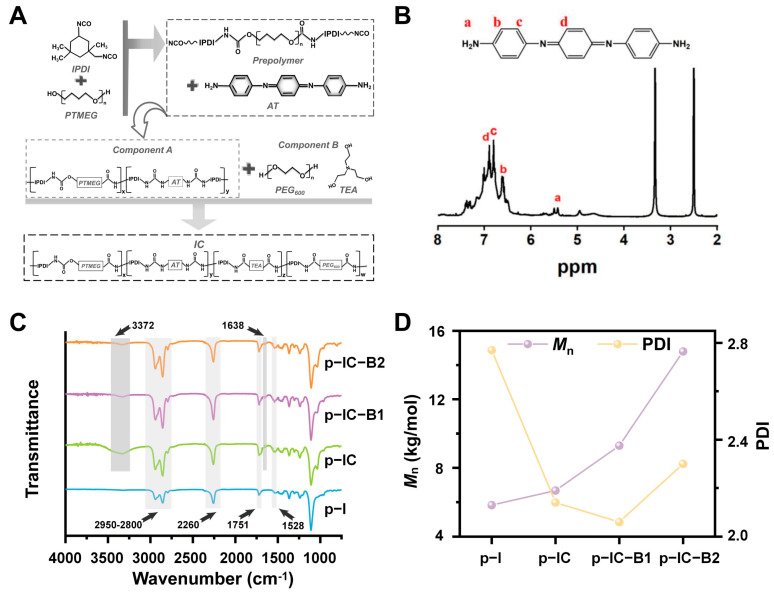
**Synthesis and Characterization of CPU-Based Prepolymers:** (**A**) Reaction scheme for synthesizing conductive polyurethane (CPU) prepolymers. (**B**) ^1^H NMR spectrum of the synthesized aniline trimer (AT)—the peaks at 6.90 ppm (Ar-H), 6.81 ppm (Ar-H), and 5.43 ppm (-NH_2_) confirm the successful synthesis of AT. (The letters respectively represent the type of H corresponding to the peak positions on the spectral graph) (**C**) FT-IR spectra of different CPU-based pre-polymers—key functional groups, including the peak at 3372 cm^−1^ (-NHCONH-) and 1638 cm^−1^ (C=O), confirm the successful grafting of AT onto the polymer backbone. (**D**) Average molecular weight and polydispersion index (PDI) of p-I and p-IC prepolymers—the p-IC-B2 group exhibits the highest *M*_n_ (2.53 times that of p-I) and the lowest PDI (2.06), indicating enhanced homogeneity due to the incorporation of AT and n-BaTiO_3_.

The reaction diagram of the conductive polyurethane is shown in Figure 2A. As the basic segment of conductive polyurethane (CPU), AT was synthesized and the molecular structure was investigated by ^1^H NMR and FT-IR spectra. As shown in Figure 2B, the absorption peaks are 6.90 ppm (s, 2H, Ar H), 6.81 ppm (s, 2H, Ar H), and 6.62 ppm (s, 2H, Ar H), which correspond to proton hydrogen on the benzene and quinone rings in the AT structure [34]. Moreover, the absorption peak of 5.43 ppm (s, 4H, NH_2_) corresponded to the terminal amino proton peak in the AT structure [35]. As shown in the FT-IR spectra in Figure S3, three characteristic absorption peaks of 3427 cm^−1^, 3334 cm^−1^, and 3240 cm^−1^ in the range of 3427–3240 cm^−1^ were attributed to the terminal amino group (-NH_2_) in the structure of AT. The characteristic peaks indicated that amino-terminated aniline trimers (AT) had been successfully synthesized and grafted in the CPU prepolymer.

Prepolymers as component A were copolymerized by PTMEG, IPDI, and/or amine-capped AT, added with functional inorganic particles including n-HA, Bi_2_O_3_, n-ZnO, and piezoelectric n-BaTiO_3_. The FT-IR spectra of the CPU-based prepolymers in Figure 2C showed that the characteristic peaks attributed to carbamate (-NHC=O), carbonyl (C=O) on the ester group, and methylene (-CH-) appeared at 1528 cm^−1^, 1751 cm^−1^, and 2800–2950 cm^−1^, respectively. For all the CPU-based prepolymers, the characteristic peaks attributed to the benzene rings of AT were observed at 1502 and 1164 cm^−1^ in Figure S3, indicating the successful linkage of AT to the backbone of conductive prepolymers (IC and IC-B). After copolymerization, the AT fragment was successfully embedded into the conductive polyurethane gels (IC, IC-B1, and IC-B2). However, the characteristic peak of the isocyanate group (-NCO) appeared at 2260 cm^−1^ in the spectra of all the prepolymers, indicating that these prepolymers contain a significant amount of active isocyanate groups. In Figure 2D, the number–average molecular weight (*M*_n_) of the p-IC group was affected by grafted AT higher than that of the p-I group, while the *M*_n_ of the p-IC-B1 and p-IC-B2 groups increased largely with the addition of n-BaTiO_3_ content. Accordingly, the *M*_n_ of the p-IC-B2 group was about 2.53 times over that of the p-I group. However, the polydispersion index (PDI) was decreased with AT and n-BaTiO_3_ added from 2.77 (p-I) down to 2.30 in the p-IC-B2 group, while the lowest value was 2.06 for the p-IC-B1 group at a narrow molecular distribution (Figure 2D). The high molecular weight and low polydispersity mean the addition of AT segment and n-BaTiO_3_ particles could help a homogenous polymeric structure forming in the CPU composites, but more nanoparticles in the p-IC-B2 group have inhibited the polymerization.

### 2.2. Physicochemical Properties of Different Composite Gels for Root Canal Therapy

**Figure 3:**
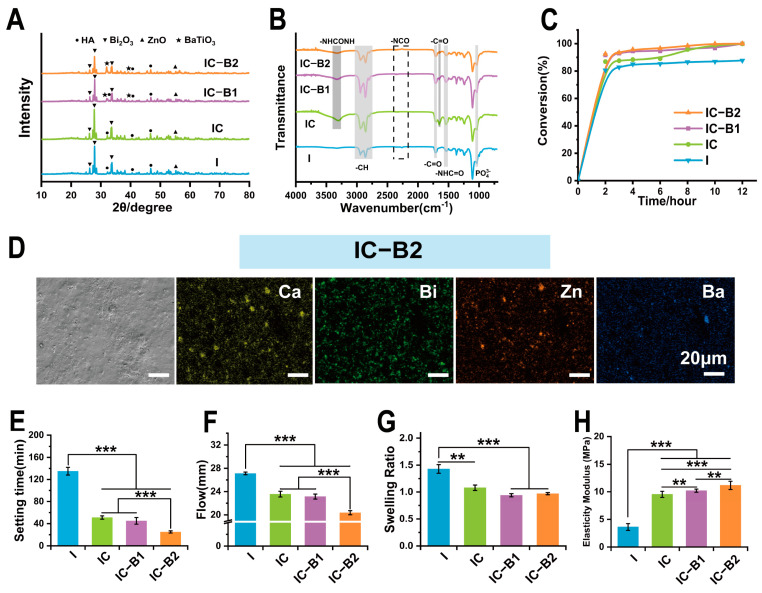
**Physicochemical properties of injectable CPU gels for root canal therapy:** (**A**) XRD and (**B**) FT-IR spectra of the cured gels. (**C**) NCO conversion degree of the different gels in 12 h. (**D**) SEM image and element distribution of Ca, Bi, Zn, and Ba on the surface of the cured IC-B2. (**E**) Setting time (*n* = 3) and (**F**) flowability (*n* = 3) of the different gels, (**G**) Welling rate (*n* = 3), and (**H**) Elastic modulus (*n* = 5) of cured gels. ** *p* < 0.01 and *** *p* < 0.001.

After being mixed, component A (prepolymers) and component B underwent a chain extension reaction to prepare injectable CPU gels. In Figure 3A, characteristic peaks corresponding to n-HA, Bi_2_O_3_, and n-ZnO appeared in the XRD patterns for all the groups of samples, which proved that the three functional nanoparticles were successfully loaded in the injectable conductive polyurethane matrix ((I, IC, IC-B1, and IC-B2)). Further, the IC-B1 and IC-B2 groups showed characteristic peaks corresponding to n-BaTiO_3_, indicating that piezoelectrical n-BaTiO_3_ was successfully loaded in the IC-B1 and IC-B2 groups.

Evidently from IR spectra in Figure 3B, the characteristic peak of isocyanate was not observed at 2260 cm^−1^ for all groups after mixing, indicating that the isocyanate groups had been completely consumed after gels curing. The NCO conversion degree has been calculated by the characteristic peak areas of FTIR spectra (at 2200–2300 cm^−1^) of gels (Figure S4A), wherein the NCO group of the IC-B2 gel decayed fast at the beginning of curing and then stabled slowly after 2 h at the conversion rate of 92.50% until 12 h at the conversion rate of 99.99% (Figure 3C and Figure S4B). Other groups presented a similar trend except the conversion rate of the I gel with only 87.69% at 12 h. Isocyanate monomers of the CPU gels (IC, IC-B1, and IC-B2 groups) were almost consumed after 12 h which is good for root canal therapy. In particular, there was a characteristic stretching vibration peak attributed to ureyl (-NHCONH-) at 3372 cm^−1^, and an asymmetric stretching vibration peak attributed to carbonyl (C=O) at 1638 cm^−1^ in the FT-IR spectra of the CPU composite gels (IC, IC-B1, and IC-B2, Figure 3B). The peaks indicated that the AT segment was successfully grafted into polymeric chains as the terminal amino group of AT was transformed into a urea group [36]. However, the three characteristic peaks of AT did not appear in the spectra of cured samples (Figure 3B), which indicates that all the terminal amino groups of aniline trimers had been transformed into the urea group. As shown in Figure 3D and Figure S5 (Supplementary Materials), the nanoparticles were uniformly dispersed in the polymeric matrix in SEM images. At the same time, the Ca, Bi, and Zn elements were all uniformly dispersed in the four groups of cured sample matrix, and the Ba element was evenly dispersed in both the IC-B1 and IC-B2 groups.

The anti-washout property of gels is an important factor in successful root canal therapy [37]. As shown in Figure S6, the pristine PU gel of the I group began to disintegrate with some dropped particles appearing after soaking for 30 min. With the time increasing, the disintegration phenomenon of the I group was more serious. In contrast, the samples of the CPU gels (IC, IC-B1, and IC-B2 groups) maintained their original shape until 4 h without any disintegration and particle shedding. The anti-washout results showed that the introduction of AT and n-BaTiO_3_ improved the anti-elution performance of the CPU gels (IC, IC-B1, and IC-B2 groups).

Setting time and fluidity are two key characteristics of root canal fillings. ISO 6876: 2012 requires the setting time of the filling to be less than 4320 min and the fluidity to be greater than 17 mm [38]. The results in Figure 3E,F showed that the setting time and fluidity of the four groups met the ISO requirements. With the introduction of AT as a chain extender, the setting time of the IC group was significantly reduced to 51 ± 3 min compared with the I group (*p* < 0.001, Figure 3E); with the increasing addition of n-BaTiO_3_, the setting time of the IC-B1 and IC-B2 groups was further reduced (*p* < 0.001), and the curing time of the IC-B2 group reached 21 ± 2 min. The fluidity of the material also showed a similar trend with the setting time in Figure 3F.

A lot of injectable biomaterials are cured by accelerating the exothermic polymerization reaction through exothermic reactions, wherein a high temperature over 47 °C for 300 s will cause severe thermal damage to tissues [39]. Here, the temperature fluctuation during the curing process was tested and recorded in a range between 34.9 °C and 36.5 °C for all the gel groups (Figure S4C, Supplementary Materials). The narrow temperature range of four PU gels testified that the series of cement could be mildly set in a biological environment without high energy release. After curing for 2 h, the isocyanate groups were diminished (Figure 3C), and the exothermic reaction stabilized (Figure S4B). Among these gels, the isocyanate conversion rate of the IC-B2 group was the highest, reaching 99.99% at 12 h.

Unlike shrinkable root canal filling, polyurethane-based materials can expand in volume after curing, providing effective obstruction within the root canal. However, inorganic particles may influence the extent of this volume expansion [40]. In Figure 3G, the swelling rates of the CPU gels (IC, IC-B1, and IC-B2 groups) were significantly reduced compared with I (*p* < 0.01 and *p* < 0.001). Meanwhile, the gel fractions of three CPU gels (IC, IC-B1, and IC-B2 groups) were higher than that of the I group in Table S1 (*p* < 0.05, Supplementary Materials). The introduction of a chain extender and inorganic particles significantly increased the crosslinking density of IC, IC-B1, and IC-B2 groups after curing. Accordingly, the elastic modulus of samples for IC, IC-B1, and IC-B2 groups have also increased by 2.6 ~ 3 times compared with that of the I group (Figure 3H, *p* < 0.01 and *p* < 0.001). For the IC-B2 group, the maximum modulus was 11.17 ± 0.75 MPa among all the gels, which is far less than the elastic modulus of dentin (16 GPa) [41]. Thus, the stress generated by volumetric expansion would not cause additional damage to the dentin.

### 2.3. Piezoelectric Response of Gels Under Cyclic Compressive Load

**Figure 4:**
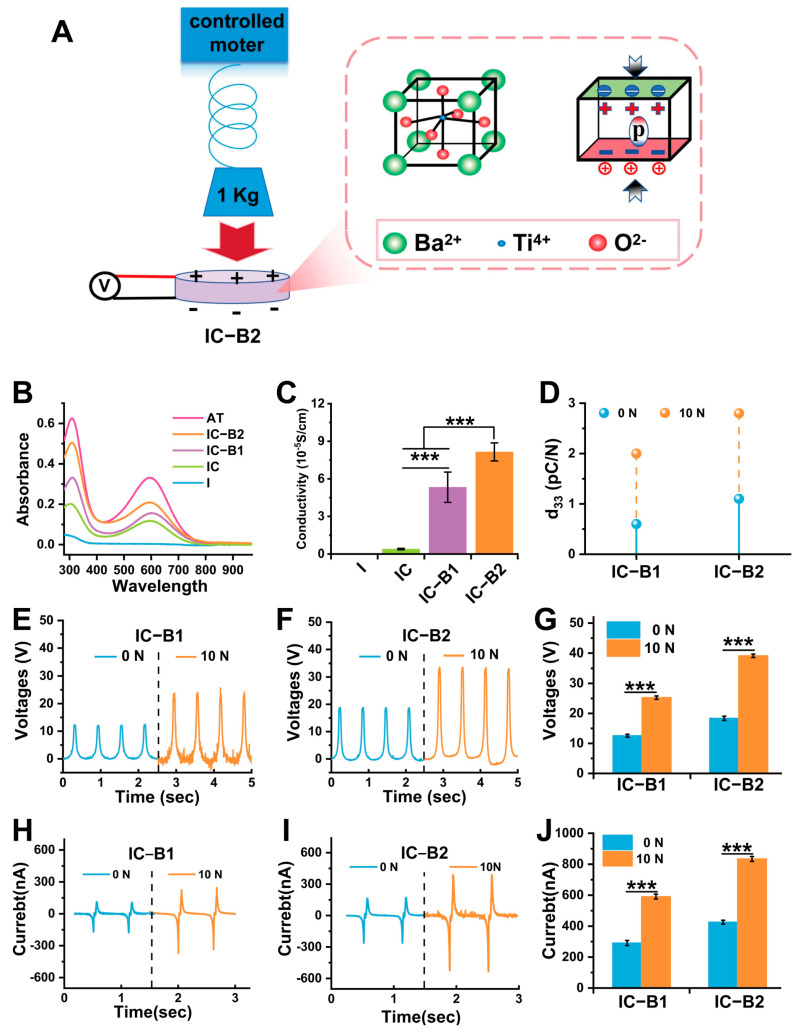
**Characterization of electrochemical properties of cured gels under 10 N compression loading for 30 cycles or unloaded condition:** (**A**) Schematic diagram of the device for compressive loading: Mechanical stress (10 N, 30 cycles) induces lattice deformation in n-BaTiO_3_, generating a piezoelectric potential. (**B**) UV-vis spectra of cured gels: The absorption peaks at 311 nm (π-π* transition) and 600 nm (quinoid exciton transition) confirm the successful integration of AT. (**C**) Conductivity of cured gels: IC-B2 achieved a conductivity of 8.15 × 10^−5^ S/cm, which is 33.8 times higher than that of non-piezoelectric control (IC group), due to AT/n-BaTiO_3_ synergy. (**D**) Piezoelectric constant of cured gels (under cyclic loading or no loading): Cyclic loading enhances the piezoelectric response by 3.3× (IC-B1) and 2.5× (IC-B2). (**E**–**G**) Output voltage spectra of IC-B1 and IC-B2 cured gels and their voltage intensities (under cyclic loading or no loading): Cyclic loading enhances the output voltage by 2.01× (IC-B1) and 2.13× (IC-B2). (**H**–**J**) Output current spectra of IC-B1 and IC-B2 cured gels and their electrical current intensities (under cyclic loading or no loading): Cyclic loading enhances the output current by 1.9× (IC-B2). (*n* = 3) *** *p* < 0.001.

Figure 4A illustrates the schematic diagram of the pressurizing device and the principle of the piezoelectric effect under compressive loading. When the sample is subjected to cyclic compressive loading, the positive and negative charge centers of the n-BaTiO_3_ filler separate, resulting in dipoles that are orderly arranged within the matrix. The action ultimately generates an electric field inside the gel. Based on the selection of cyclic compressive stress parameters, a cyclic compressive stress of 10 N was applied to the cured gels for 30 cycles (half a minute per cycle) optimized in the subsequent experiment (Figure S1).

The UV absorption spectra of the IC, IC-B1, and IC-B2 gels are shown in Figure 4B. The spectral profiles exhibited two well-resolved absorption bands at 311 nm and 600 nm, which were, respectively, attributed to the characteristic π-π* electronic transition within the aromatic system and the intermolecular charge-transfer exciton transition occurring between the benzenoid and quinonoid moieties [42]. The two peaks did not appear in the spectrum of the I gel. The exciton transition from the benzene ring to the quinone ring, corresponding to the absorption peak at 600 nm, can be attributed to the successful incorporation of the AT fragments into the chain segments of the IC, IC-B1, and IC-B2 gels. This incorporation ameliorated the polymeric structure, thereby enhancing electrical activity [43]. Accordingly, the conductivity of the I gel could not be measured within the detection range of the instrument in Figure 4C. The conductivity of the IC gel reached 3.92 ± 0.38 × 10^−6^ S/cm. In particular, the conductivity values of the IC-B1 and IC-B2 gels were in the same order of magnitude of 5.33 ± 0.44 × 10^−5^ S/cm and 8.15 ± 0.75 × 10^−5^ S/cm, respectively, higher than that of the IC gel (*p* < 0.001). With the increase in n-BaTiO_3_ content, the conductivity of the IC-B2 gel increased to 1.5 times over than that of IC-B1 (*p* < 0.001).

Under different compressive loading conditions (0 N and 10 N), Figure 4E,F show the representative voltage generated by the cured gels IC-B1 and IC-B2, while Figure 4H,I show the representative current generated by the cured gels IC-B1 and IC-B2. From the quantified results in Figure 4G, without external force loading, the output voltage intensity of the IC-B2 gel was 1.46 times higher than that of IC-B1 (12.56 ± 0.19 V, *p* < 0.001). After applying a compressive load of 10 N for 30 cycles (half a minute per cycle), the output voltage of IC-B1 and IC-B2 had been significantly improved. Among them, IC-B1 under cyclic loading increased to 2.01 times the output voltage higher than the original without loading, and IC-B2 increased to 2.13 times over the original with the output voltage reaching 39.14 ± 0.53 V (*p* < 0.001, Figure 4G). The output current intensity of IC-B1 and IC-B2 also showed a similar enhancement trend as output voltage intensity, with IC-B2 exhibiting a 1.9-fold increase under cyclic loading compared to the original under 0 N, reaching 834.34 ± 16.05 nA (*p* < 0.001, Figure 4J). The piezoelectric constant of IC-B2 was 1.83 times over that of IC-B1 without cyclic loading (Figure 4D). Under the cyclic loading, the piezoelectric constant of IC-B1 increased by 3.33 times than without loading, and the piezoelectric constant of IC-B2 increased by 2.54 times than without loading, reaching 2.8 pC/N (Figure 4D). These electrical characteristics showed that the cyclic loading could significantly improve the electroactivity of the BaTiO_3_-added composite gels (IC-B1 and IC-B2 groups).

### 2.4. Antibacterial Properties of Gels Under Cyclic Loading

**Figure 5:**
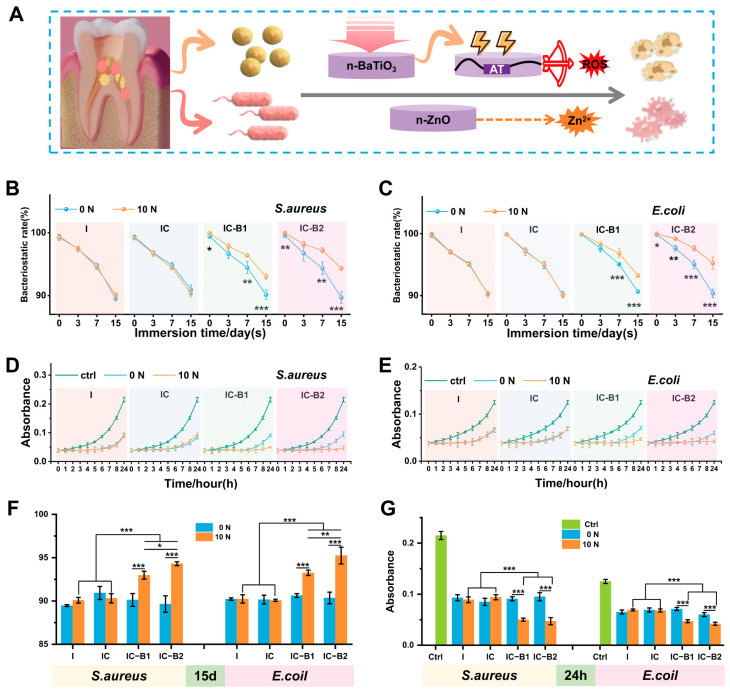
**Bacteriostasis properties of cured gels under 10 N compression loading for 30 cycles or unloaded condition:** (**A**) Antibacterial diagram of different bacteria. (**B**) Antibacterial rates from the static contact test (SCT) over 15 days against *S. aureus* and (**C**) *E. coli.* (**D**) Dynamic contact test curves (DCT) of the various groups after 15 days of soaking in PBS solution against *S. aureus* and (**E**) *E. coli*. (**F**) Antibacterial rates from the SCT over 15 days against *S. aureus* and *E. coli.* (**G**) After 15 days of soaking in PBS solution, the absorbance of DCT of each group against *S. aureus* and *E. coli* at 24 h. The uncoated wells were used as the blank control group. * *p* < 0.05, ** *p* < 0.01, and *** *p* < 0.001 (*n* = 3).

Both Gram-positive *S. aureus* and Gram-negative *E. coli* were selected as bacterial models to test the broad-spectrum antibacterial effect of the injectable CPU gels (Figure 5A). Figure 5B,C and Figure S7A,B show the inhibition rate of the tested sample against *S. aureus* and *E. coli* directly in a static contact test (SCT). In the static contact experiment with bacteria, the antibacterial ability of the four groups was impaired as the soaking time increased (Figure S7A,B). After 15 days of soaking, the antibacterial rate of all the gels decreased to approximately 90% under unloaded conditions. However, when the axial compressive load of 10 N was applied for 30 cycles, the antibacterial rate of IC-B1 and IC-B2 significantly improved compared to the original unloaded groups from the first day. In contrast, no change was observed in the I and IC groups (Figure 5B,C). Especially, the antibacterial rate of IC-B2 against *S. aureus* and *E. coli* increased to approximately 95% after 15 days of soaking. After 15 days of soaking, the antibacterial rate of the IC-B1 group increased significantly by 2.86%, while that of the IC-B2 group increased significantly by 4.76%, which was 1.66 times higher than that of the IC-B1 group (*p* < 0.01 and *p* < 0.001, Figure 5F). The antibacterial efficacy of the four groups of materials against *E. coli* was similar to that against *S. aureus*. After soaking for 15 days, the antibacterial rate of IC-B2 increased by 5.25% under cyclic loading (*p* < 0.001, Figure 5F).

According to the data obtained from the Dynamic Contact Test (DCT) for *S. aureus* and *E. coli* in Figure 5D,E and Figure S7C,D, the four groups of gels significantly inhibited the proliferation of *S. aureus* and *E. coli* in the first 6 h after soaking for 15 days. The inhibitory effects of the I and IC groups did not change after the application of pressure. The IC-B1 group had a significant inhibitory effect in the first 8 h, while the IC-B2 group effectively inhibited *S. aureus* and *E. coli* for up to 24 h. The DCT curves showed that the mechano-powered CPU gels exhibited a good antibacterial effect. Under the cyclic compressive loading, the antibacterial effect of the IC-B1 and IC-B2 groups after 15 days soaked in PBS was significantly increased to 24 h (from *p* < 0.01 to *p* < 0.001, Figure 5G). It can be postulated that mechano-powered gels could exhibit an excellent and sustained antibacterial effect under the frequent chewing action in the oral cavity.

### 2.5. The Ability of Gels to Eradicate Bacterial Biofilms Under Cyclic Loading

**Figure 6:**
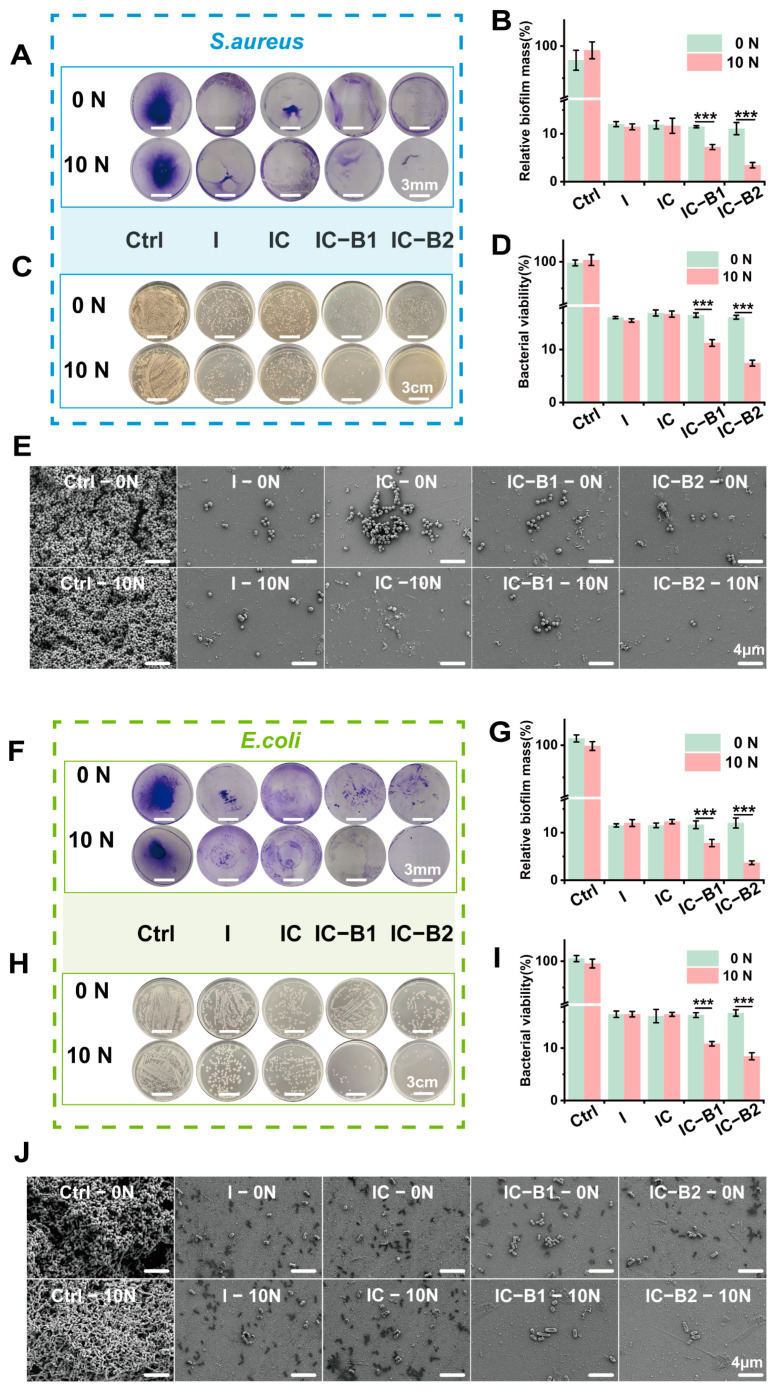
**In vitro biofilm eradication and bacterial killing efficiency of cured gels under 10 N compression loading for 30 cycles or unloaded condition:** (**A**) Crystal violet staining image of *S. aureus* biofilm (the chromatic intensity of the purple-stained solutions provides a quantitative chromogenic measure of retained biofilm biomass). (**B**) Semi-quantitative analysis of crystal violet staining intensity for residual *S. aureus* biofilm. (**C**) Agar plate count of bacterial viability in *S. aureus* biofilm. (**D**) Quantified results of bacterial viability within the *S. aureus* biofilm. (**E**) SEM images of *S. aureus* residue biofilm (×10,000). (**F**) Crystal violet staining image of *E. coli* biofilm (The chromatic intensity of the purple-stained solutions provides a quantitative chromogenic measure of retained biofilm biomass). (**G**) Semi-quantitative analysis of crystal violet staining intensity for residual *E. coli* biofilm. (**H**) Agar plate count of bacterial viability in *E. coli* biofilm. (**I**) Quantified results of bacterial viability within *E. coli* biofilm (**J**) SEM images of *E. coli* residue biofilm (×10,000). *** *p* < 0.001. (*n* = 3).

Bacterial biofilm is a self-protection mechanism that bacteria develop during their process. The formation of biofilm hinders the interaction between bactericidal substances and bacteria, thereby reducing the efficacy of these bactericidal agents [44]. Figure 6A shows a crystal violet staining image of a *S. aureus* biofilm, where the stained crystal violet was dissolved in ethanol and the absorbance measured reflects the level of residual biofilm. Figure 6B presents a semi-quantitative analysis of the residual biofilm level. The efficiency of biofilm eradication for the four gel groups was maintained at 88% under pressure-free conditions. After cyclic compressive loading, the biofilm eradication rate for the IC-B1 group was significantly improved to 92% compared to the original groups without loading (*p* < 0.001), and the biofilm eradication rate for IC-B2 was also significantly improved to 96% (*p* < 0.001). Figure 6C,D show agar plate images of bacterial colony counts in residual biofilms, as well as a quantitative analysis of bacterial viability. In the absence of loading pressure, 15% of the bacteria survived; after cyclic loading, the survival rate decreased to 10% for IC-B1 and 7% for IC-B2 (*p* < 0.001). Figure 6F–I show that the eradication ability of the material against the *E. coli* biofilm was similar to that of the *S. aureus* biofilm. After applying cyclic loading, the eradication rate of the *E. coli* biofilm for IC-B2 increased to 97%, and the survival rate of bacteria within the membrane decreased to 7% (*p* < 0.001).

Figure 6E,J show the SEM morphology of the residual *S. aureus* and *E. coli* biofilms. The original biofilm was dense and thick; the bacteria in the *S. aureus* biofilm remained typically spherical, while those in the *E. coli* biofilm remained typically rod-shaped. Under cyclic loading, only sparse bacterial biofilms were observed in both the IC-B1 and IC-B2 gels. In Figure 6E,J, the bacterial surface appeared wrinkled and contracted, with some cells losing membrane integrity and leaking cellular contents. In contrast to the two piezoelectric-enhanced gels (IC-B1 and IC-B2), both the I and IC groups still retained a small amount of biofilm regardless of loading or not, even if the chemical fungicide (n-ZnO) and electroactive component (AT) were incorporated in the composite gels (Figure 6E,J).

### 2.6. Chemical Structure Changes and ROS Generation of Gels Under Cyclic Loading

**Figure 7:**
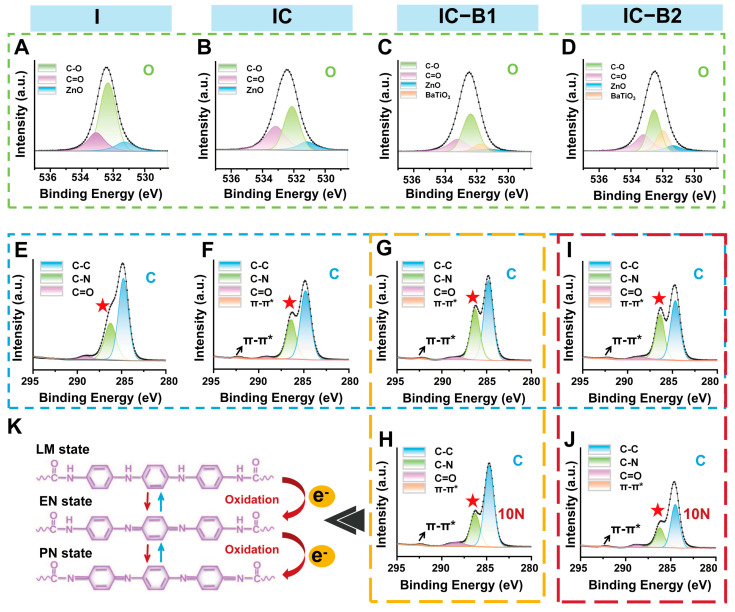
**Deconvoluted XPS spectra of cured gels under 10 N compression loading for 30 cycles or unloaded condition:** High-resolution XPS spectra of (**A**) O 1s in I gel; (**B**) O 1s in IC gel; (**C**) O 1s in IC-B1 gel; (**D**) O 1s in IC-B2 gel; (**E**) C 1s in I gel; (**F**) C 1s in IC gel; C 1s in IC-B1 gel (**G**) under no load and (**H**) under cyclic loading; C 1s in IC-B2 gel (**I**) under no load and (**J**) under cyclic loading. (**K**) Schematic diagram of chemical structure change in AT fragment in CPU matrix under loading. Red star: C-N bond at 286 eV; black arrow: the π-π* peak at 292 eV.

To investigate the effect of cyclic loading on the composite gels, high-resolution XPS spectra of O 1s were deconvoluted to analyze the oxygen-related bonding states (Figure 7A–D). In the fitted graph, the peak of n-BaTiO_3_ was observed in both the IC-B1 and IC-B2 gels, confirming the successful incorporation of n-BaTiO_3_ into the polymeric matrix. High-resolution XPS spectra of gels C-1s were deconvolved to analyze key carbon states. For the C 1s spectra (Figure 7E–J), the peak at 292 eV in the IC gel (with AT as a chain extender) corresponds to the π-π* transition within the conjugated aromatic system of the benzene ring. Additionally, the increased intensity of the C-N bond peak at 286 eV in the IC gel indicates the successful integration of AT into the polyurethane backbone (red star, Figure 7F), as compared to the control (I gel, Figure 7E). After cyclic loading, the peak intensity of the C-N bond (286 eV, red star) in the C 1s spectra of the IC-B1 gel (Figure 7H) was observed to decrease by 21.78% compared to that in the IC-B1 gel without loading (Figure 7G). Under the same mechanical conditions, the π-π* peak (292 eV) of IC-B1 remained unchanged regardless of whether loading was applied (black arrow). In particular, the peak intensity corresponding to the C-N bond (286 eV) in IC-B2 decreased by 48.34% after applying cyclic pressures. The π-π* (292 eV) peak remained stable, confirming the structural integrity of the aromatic system (Figure 7I,J). The changes in the C-N bond related to grafted-AT in PU suggest that IC-B2 is more sensitive to mechanical stress than IC-B1, leading to greater structural change.

During the oxidation process of AT fragments in polyurethane, electrons were produced and transferred to surrounding H_2_O and O_2_, leading to the formation of ROS such as hydroxyl radicals (•OH) and superoxide anions (•O_2_^−^). The level of generated ROS is crucial in disrupting bacteria metabolism and biofilm formation [45]. So, the ability and types of ROS generation of the injectable and conductive polyurethane gels were assessed according to a previous study [46].

**Figure 8:**
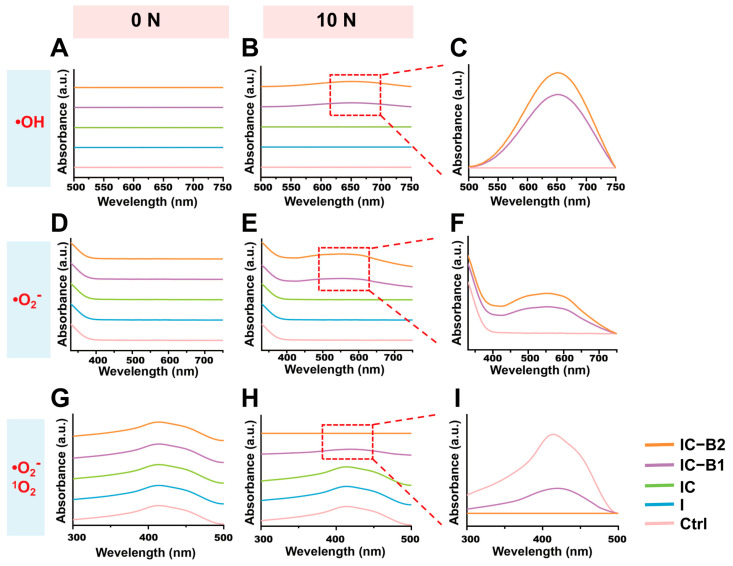
**ROS generation performed by UV-vis spectra for gels under 10 N compression loading for 30 cycles or unloaded condition:** (**A**) The UV-vis spectra of TMB were used to characterize the ·OH produced by four groups of conductive polyurethanes without pressure. (**B**) The UV-vis spectra of TMB were used to characterize the ·OH produced by four groups of conductive polyurethanes under cyclic pressure. (**C**) The UV-vis spectra of TMB were used to characterize the ·OH produced by IC-B1 and IC-B2 under cyclic pressure. (**D**) The UV-vis spectra of NBT were used to characterize the ·O_2_^−^ produced by four groups of conductive polyurethanes without pressure. (**E**) The UV-vis spectra of NBT were used to characterize the ·O_2_^−^ produced by four groups of conductive polyurethanes under cyclic pressure. (**F**) The UV-vis spectra of NBT were used to characterize the ·O_2_^−^ produced by IC-B1 and IC-B2 under cyclic pressure. (**G**) The Uv-absorbable spectra of DPBF were used to characterize the ^1^O_2_/O_2_^−^ produced by four groups of conductive polyurethanes without pressure. (**H**) The Uv-absorbable spectra of DPBF were used to characterize the ^1^O_2_/O_2_^−^ produced by four groups of conductive polyurethanes under cyclic pressure. (**I**) The Uv-absorbable spectra of DPBF were used to characterize the ^1^O_2_/O_2_^−^ produced by IC-B1 and IC-B2 under cyclic pressure. Ctrl group: No sample was placed in the indicator solution, and other conditions were consistent with those of the experimental group.

The colorless TMB could react with •OH to form blue oxidized TMB (oxTMB) with a typical absorption peak at 652 nm. In Figure 8A, no peak has been observed in all the groups without loading. Under cyclic loading, a characteristic peak at 652 nm was observed only in the IC-B1 and IC-B2 groups, whereas other groups showed no detectable peak at this wavelength (Figure 8B,C). The peak observed at 652 nm in both the IC-B1 and IC-B2 groups confirmed the generation of hydroxyl radicals (•OH) under loading conditions. As the number of loading cycles increased, the intensity of peak at 652 nm gradually increased (Figure S1A,B), demonstrating the time-dependent production of •OH for both the IC-B1 and IC-B2 gels. In addition, during the same cycle period, the intensity of the peak at 652 nm of IC-B2 was 129.59% higher than that of IC-B1 under 30 cycles loading, which indicates that IC-B2 gel generated more hydroxyl radicals (•OH) under cyclic loading than that of IC-B1 (Figure S1C). For •O_2_^−^ detection, NBT was reduced to blue formazan, monitoring by its absorption peak at 560 nm [46]. Similarly to the formation of •OH, only IC-B1 and IC-B2 exhibited obvious absorption peaks at 560 nm under cyclic pressure and the peak intensity at 560 nm increased with increasing the loading cycles, confirming the generation of •O_2_^−^ as a time-dependence mode (Figure 8E,F and Figure S1D,E). Under the same cycle of loading conditions, the characteristic peak intensity at 560 nm for IC-B2 was 150.72% higher than that of IC-B1, indicating that IC-B2 generated more superoxide anions than that of IC-B1 after 30 loading cycles (Figure S1F). DPBF is an indicator with a characteristic peak at 420 nm. Upon reacting with ^1^O_2_ and •O_2_^−^, yellow DPBF forms endoperoxides and transforms into colorless 1, 2-dibenzoylbenzene, causing the characteristic absorption peak at 420 nm to weaken or disappear [47]. Under cyclic loading, the characteristic peaks of IC-B1 and IC-B2 at 420 nm weaken and disappear, confirming the generation of ^1^O_2_ and •O_2_^−^ (Figure 8H,I). As the cycle period increased, the absorption intensity at 420 nm gradually decreased, indicating that the production of ^1^O_2_ and •O_2_^−^ is time-dependent (Figure S1G,H). Moreover, within the same cycle period, the absorption peak intensity at 420 nm for the IC-B2 gel decreased by 153.03% more than that of IC-B1, indicating that a high sensitivity to ^1^O_2_ and •O_2_^−^ generation in the IC-B2 group compared to IC-B1 (Figure S1I).

### 2.7. Biocompatibility of Gels Under Cyclic Loading

**Figure 9:**
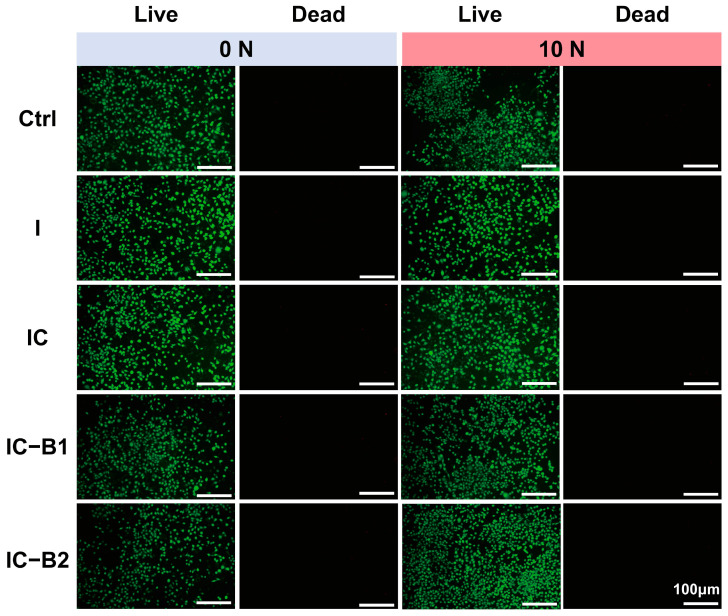
Biocompatibility assessment of the polyurethane gels under mechanical loading. The staining of live/dead cells was performed after culturing L929 cells in the polyurethane gel extract for 7 days under two conditions: (1) 10 N compression loading applied for 30 cycles, or (2) an unloaded condition. In the loaded condition, one group had the culture solution in contact with the solid material under pressure, while the other group remained untreated without additional operations. Green: living cells; red: dead cells).

The proliferation of L929 cells was measured using cck-8. When subjected to a compressive load of 10 N for 30 cycles, the mechano-powered gels (IC-B1 and IC-B2) generated a current that could be transmitted through the wire to the pore plate of the cultured cell (Figure S8A). Figure S8B shows that each group of cells exhibited a tendency to proliferate as the culture time extended. In the absence of loading, the OD value of materials in each group was slightly lower than that in the control group with no significant difference (Figure S8B). The cell viability shows that all the gels had good biocompatibility. According to the aforementioned working mechanism, the IC-B2 group exhibited the highest cell viability, which was significantly higher than that of the original groups without loading from 1 day (*p* < 0.01) to 7 days (*p* < 0.001), while the IC-B1 group significantly proliferated from 4 d to 7 days (*p* < 0.05). Simultaneously, the cell proliferation in groups I and IC remained unchanged regardless of whether pressure loading was applied. The staining results of live/dead cells presented in Figure 9 confirm the biocompatibility of all four gel groups. Specifically, the IC-B1 and IC-B2 gels exhibit enhanced L929 cell proliferation under loading conditions, attributable to the bioelectric currents generated by their piezoelectric activity. Critically, these gels show no cytotoxic effects, as evidenced by high cell viability and minimal cell death in the stained assays.

### 2.8. Discussion

The frequent chewing behavior in daily life generates significant compressive stress within the oral environment [13]. Under the influence of this stress, an electric field is induced on the surface of piezoelectric materials [25]. The mouth, acting as a natural energy source, can be harnessed to power these piezoelectric materials. The conversion of mechanical energy into electrical effects ensures sustained antibacterial efficacy against recurrent infections in the complex oral environment. To address the limited lifespan of traditional antibacterial agents, a mechano-powered strategy has been developed to achieve prolonged bacteriostasis. This approach utilizes an injectable and conductive polyurethane composite gel for root canal therapy. In this study, piezoelectric n-BaTiO_3_ and antimicrobial n-ZnO were synergistically combined with conductive AT segments in a polyurethane matrix to create a piezoelectric antibacterial gel. Within this system, an integrated piezoelectric/conductive network was constructed to facilitate efficient charge transport via the mechano-electric coupling effect. Powered by the piezoelectric/conductive matrix, the conductivity of the composite gels was significantly enhanced under cyclic compressive loading (Figure 4). Building upon this piezoelectric enhancement, the antibacterial properties of the gels under pressure-induced activation were further investigated. Focusing on the protection mechanism against bacterial biofilms, the mechano-powered gel was dynamically activated through cyclic loading, leveraging the mechano-electric coupling effect to effectively eradicate biofilms in the oral environment.

#### 2.8.1. Electro-Responsive Behavior of the Conductive Gels Under Cyclic Loading

The human body contains abundant untapped biomechanical energy that could potentially be harvested and transformed into therapeutic electrical signals through specialized energy conversion processes. Everyday human activities provide numerous opportunities for energy harvesting, such as the mechanical energy generated by chewing in the mouth. A promising way is to harvest the “intermittent motion” energy by using piezoelectricity [48]. Piezoelectric substances demonstrate intrinsic electromechanical coupling through spontaneous electrical potential generation upon mechanical deformation [49]. The incorporation of AT segments formed urea bonds with the polyurethane matrix, enhancing crosslinking density and mechanical strength (reduced swelling and higher modulus). Synergy with n-BaTiO_3_ via hydrogen bonding created a multiscale network, optimizing both mechanical resilience and electrical performance, consistent with prior studies [31,50]. After the AT segment was successfully grafted onto the molecular chain of polyurethane, the conductive polyurethane performed a typical electric response (Figure 4B,C). The CPU polymeric matrix could form a continuous conductive network providing high electro-sensitivity for the mechano-powered gel, efficiently transferring the charges transduced by the piezoelectric effect, and playing a positive role in the mechano-electric coupling effect on the dynamic antibacterial activity of the CPU gels (Figure 5 and Figure 6). Even without mechanical loading, both the IC-B1 and IC-B2 groups demonstrated significantly enhanced conductivity, with values 21.09 and 33.81 times higher than the IC group (without n-BaTiO_3_) (Figure 4C, *p* < 0.001).

The piezoelectric phenomenon fundamentally originates from atomic lattice rearrangement induced by applied mechanical forces, governed by the material’s intrinsic lattice asymmetry [50]. Mechanical strain induces lattice symmetry breaking through charge center displacement in crystalline structures, triggering spontaneous dipole formation via electrostatic imbalance that ultimately drives charge displacement along potential gradients. According to the literature, the average chewing force of human premolars is about 10 N which frequently produces a cyclic pressure in daily life [51]. So, the compressive load of 10 N was applied as the power source for the piezoelectric n-BaTiO_3_, which was the transducer in the CPU gel (IC-B1 and IC-B2). After applying cyclic compressive stress for 30 cycles to the cured samples, the output voltage and output current of the mechano-powered gels were significantly improved. Among them, the output voltage of the IC-B2 group was increased to 39.14 ± 0.53 V and the current was 834.34 ± 16.05 nA, significantly higher than that of the original gel without loading (Figure 4G,J, *p* < 0.001). In comparison, Kim added 20 wt% n-BaTiO_3_ to the insulated polyurethane substrates, and the maximum output voltage was only 2.16 V under pressure from the weight of an adult male [52]. Moreover, the piezoelectric constants of IC-B1 and IC-B2 were increased by 3.33 times and 2.54 times, respectively, after applying cyclic stress lasting 30 cycles (Figure 4D). In daily life, the chewing action is more frequent with a long-time working that could excite the dynamic antibacterial activity via the mechano-electric coupling effect of the conductive gels.

Different from the piezoelectric BaTiO_3_, n-ZnO is a widely used n-type semiconductor contributing to the presence of intrinsic defects, such as oxygen vacancies and zinc interstitials [53]. But, the pure ZnO exhibits limited electron conductivity due to its wide bandgap of approximately 3.37 eV at room temperature. The surface conductivity of n-ZnO enables efficient electron transport through doped heterojunctions [54], thermal excitation [55], and exposure to oxidizing gases (O_2_ and NO_2_) or reducing gases (H_2_ and CO) [56]. In the composite gels, no method or dopants can enhance the charge carrier mobility to introduce free electrons into the conduction band of ZnO particles. Consequently, the n-ZnO component in the I group did not display any electrical properties which were testified by the UV-spectra and conductivity values (Figure 4B,C). Under the same test condition, the conductivity of the IC group also cannot be strengthened under the mechanical loading for the chemical structure of AT conductive molecules, same as the low intrinsic conductivity of the ZnO nanoparticles. For both the I and IC groups (without n-BaTiO_3_), piezoelectric characteristics could not be measured, regardless of external force loading.

At this point, the cyclic loading induces chemical structural changes in the CPU gels (IC-B1 and IC-B2). The phenomenon is attributed to the piezoelectric field generated by the n-BaTiO_3_ nanoparticles under mechanical stress. AT has different redox states similar to that of polyaniline, and redox states could be exchanged under different voltages and redox actions [57]. Therefore, the electric field facilitates the oxidation of C-N bonds to C=N bonds within the AT-grafted polyurethane chains (Figure 7K). The higher n-BaTiO_3_ content in IC-B2 amplifies this effect, as the intensified piezoelectric potential drives greater electron transfer, further reducing C-N bonds and enhancing the conjugation within the polymer backbone [31]. The increased conjugation aligns with the elevated conductivity observed in IC-B2 (Figure 4C), as extended π-electron delocalization promotes charge carrier mobility [50]. The preservation of π-π* transition peaks (Figure 7F) confirms that the aromatic structure of AT remains intact, ensuring sustained electronic communication across the conductive network [34,35]. Such structural evolution under cyclic loading establishes a synergistic piezoelectric/conductive pathway, where the mechano-electrical coupling not only amplifies charge generation but also stabilizes the redox-active states of AT, similar to polyaniline systems [57]. This dynamic interplay between piezoelectric polarization and conductive polymer redox activity underpins the enhanced electroactivity, enabling efficient charge transduction for prolonged antibacterial efficacy. These findings align with prior studies on redox-active polymers, where structural modifications via electric fields significantly modulate charge transport and interfacial interactions [31,50].

#### 2.8.2. Dynamic Antibacterial Activity of the Gels Under Cyclic Loading

Recent studies have demonstrated that piezoelectric materials, when mechanically or otherwise excited, can produce electric effects such as ROS generation, which contribute to their antibacterial properties [58,59,60]. The mechano-powered antibacterial mechanism provided a promising approach for developing piezoelectric antibacterial gel in root canal therapy. Root canal fillers are subjected to the stress of frequent tooth occlusion during service, but few design consideration is postulated by using the teeth generating pressure. Therefore, the piezoelectric n-BaTiO_3_ filler is added as the transducer of the CPU gel (IC-B1 and IC-B2) and the response sensitivity of the gels is highly excited by the bite of the teeth. Based on the natural source of stress, the mechanical energy has been converted into electrical energy, achieving the sustained killing effect of bacteria in a complex oral environment.

The first key finding of the antibacterial activity is that the decreasing trend of SCT and DCT values was not affected by the addition of n-ZnO and AT in the composites, whether cyclic loading was applied or not, even as the soaking time increased (I and IC groups, Figure 5 and Figure S7). The data show that n-ZnO played an antibacterial role in all four gel groups in the absence of pressure. Several investigations have discovered the decreasing size of ZnO nanoparticles can enhance their antibacterial efficacy [61]. Based on our previous study, n-ZnO acts as a chemical fungicide in injectable PU-based root canal filling by releasing Zn^2+^ in comparison to the bacteriostatic rate of commercial AH Plus^®^ and Apexit^®^ Plus which imploded to about 30% at 15 days [24]. Unfortunately, the gradient ions release of n-ZnO from the surface of cured root canal filling will be fading in the dry condition of a root canal, while the ROS generation of ZnO also is weakened without light irradiation in the anaerobic and darkness conditions.

To overcome the short life of chemical antibacterial agents and avoid the overuse of antibiotics, a sustained strategy has been designed by virtue of refreshed power generated by tooth occlusion. But, the antibacterial activity of n-ZnO (I group) was not affected by different compressive conditions (Figure 5), in that n-ZnO as an n-type oxide semiconductor could not transfer the mechanical press to electrical energy (Figure 4). Similar phenomena were observed for the addition of electroactive AT (IC group) shown in Figure 5. Therefore, n-BaTiO_3_ as a piezoelectric filler has been introduced in the injectable CPU gels to stimulate dynamical antibacterial activation under intermittent loading. Under cyclic loading, the piezoelectric n-BaTiO_3_ of the mechano-powered gels sustained a significantly strong antibacterial activity (IC-B1 and IC-B2 groups, 95%) for 15 days (*p* < 0.001). The antibacterial way could make full use of the natural stress generated by mouth to play an antibacterial role while avoiding the production of bacterial resistance [62].

Bacterial biofilms are key contributors to oral infections. These complex communities of microorganisms develop on various surfaces—both living (biotic) and non-living (abiotic). Once formed, the bacteria become embedded within a protective matrix, making them more resistant to viral attacks. Bacteria in biofilms are more resistant to antibiotics than their isolated confreres; especially, both living and dead biofilms present a risk for the pharmaceutical product. The community-like microbial lifestyle helps them survive longer under challenging conditions such as nutrient deficiencies, dryness, unfavorable temperatures, exposure to ultraviolet radiation, and pH, as bacterial cells embedded in biofilms are up to 1000 times more resistant [63]. Due to the existence of biofilms, traditional antimicrobial methods are insufficient in dealing with the challenges of harmful bacteria [64]. As shown in Figure 6A–I, the material eradicated approximately 90% of the biofilm under no pressure, with a bacterial survival rate within the membrane of about 15%. After applying cyclic pressure for 30 cycles, the biofilm eradication efficiency of the IC-B1 and IC-B2 groups was significantly enhanced (*p* < 0.001). Notably, the IC-B2 group demonstrated the highest efficacy, reducing the biofilm to approximately 3%, while the bacterial survival rate dropped from 15% to 7%. The exceptional antibacterial performance of the piezoelectric IC-B2 gel meets stringent clinical requirements for root canal therapy, which necessitates >90% biofilm eradication and <10% bacterial survival to prevent reinfection [9,12]. As systematically summarized in Table S3, conventional root canal materials exhibit significant limitations: Epiphany SE provides only transient antibacterial efficacy (<1 week) and lacks detectable biofilm resistance under static conditions [65,66]; Pulp Canal Filling EWT and EndoSequence BC fail to maintain antibacterial activity beyond one week and lack documented biofilm inhibition capabilities [10]. Of particular concern is AH Plus^®^/Apexit^®^ Plus, which shows a dramatic decline in antibacterial efficacy to ~30% after 15 days under static loading, with no biofilm resistance [24]. While Shah’s BaTiO_3_ nanoparticles achieve ~85 ± 3.5% biofilm resistance against *S. aureus* under static conditions [59], tour IC-B2 gel achieves a biofilm resistance rate of ~97% against both pathogens under cyclic loading (10 N, 30 cycles), surpassing the clinical threshold (>90% biofilm eradication) [9,12].

**Figure 10:**
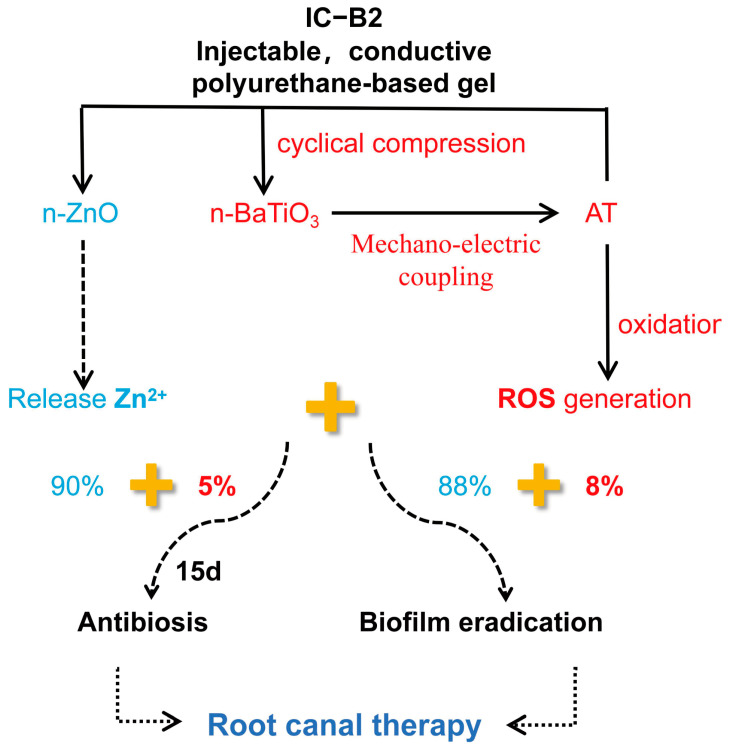
Mechanical and electrical coupling effect combined with chemical antibacterial activity to achieve root canal therapy.

The electrochemical inhibition of bacteria is a promising and robust approach currently under investigation for both the degradation and formation of biofilm. High temperatures can destroy biofilms, but the approach is not safe to deeply use inside the delicate interior of a root canal. Studies indicate that the combination of low concentrations of antibacterial agents and direct current (DC) results in a synergistic effect, significantly enhancing biofilm degradation [12]. This study presents a novel piezoelectric-enhanced strategy, integrating mechano-electric coupling effect with chemical antimicrobial activity to enable sustained biofilm eradication through dynamic antibacterial effects (Figure 10). Under cyclic compressive loading, the lattice of piezoelectric nanoparticles (n-BaTiO_3_) underflows asymmetrical deformation, leading to the displacement of positive and negative charge centers and the subsequent generation of piezoelectric potential [67]. Under cyclic loading, the repeated deformation of n-BaTiO_3_ produces alternating surface charges, achieving sustained charge transfer [31]. After grafting, the aniline trimer (AT) preserved the complete π-π* conjugated structure in the polyurethane-based conductive polymer (Figure 7F), synergistically forming a continuous piezoelectric/conductive network with n-BaTiO_3_. The interconnected network functions as an “electron highway”, facilitating efficient carrier migration and establishing a stable internal electric field. Similarly to polyaniline, AT exhibits multiple redox states that can be reversibly interconverted under applied voltage or redox stimulus [57]. Driven by the internal electric field, the benzene rings in AT undergo oxidation (with the partial conversion of C-N to C=N bonds), releasing electrons into the surrounding environment (Figure 7K). These electrons subsequently react with ambient H_2_O and O_2_ to generate abundant reactive oxygen species (ROS), including hydroxyl radicals (•OH), singlet oxygen (^1^O_2_), and superoxide anions (•O_2_^−^), of which the characteristic peaks were observed in the UV-vis spectra in Figure 8. The generating ROS exerts oxidative stress to disrupt bacterial cell membranes, degrade biofilm matrix components (polysaccharides and proteins), and damage genetic material, thereby effectively eradicating biofilms and inhibiting bacterial proliferation [45]. Simultaneously, the electric field has enhanced ROS interaction with negatively charged bacterial surfaces through electrostatic attraction (Figure 6E,J), amplifying antibacterial efficacy (Figure 5). Crucially, the dynamic mechanical stimulation from natural oral chewing can sustainably promote ROS generation through the piezoelectric–electrochemical coupling mechanism, enabling the long-term targeted disruption of biofilm regeneration and reactivation. Although ROS was detected under cyclic loading (Figure 8B,F,H), CCK-8 and Live/Dead assays confirmed no detectable cytotoxicity (Figure S8B and Figure 9). This selective antibacterial effect arises from fundamental differences in oxidative stress defense between bacteria and mammalian cells. Unlike mammalian cells, bacteria lack sophisticated ROS-neutralizing systems such as superoxide dismutase (SOD), catalase (CAT), and glutathione peroxidase (GPx) [68,69]. For instance, *S. aureus* and *E. coli* depend on limited enzymatic pathways, such as alkyl hydroperoxide reductase AhpCF [70], making them highly vulnerable to oxidative stress. In contrast, mammalian cells possess robust antioxidant networks capable of efficiently neutralizing moderate ROS levels [69]. The finding aligns with prior studies showing that piezoelectric materials can generate therapeutically useful ROS concentrations without compromising mammalian cell viability [63]. The mechano-electrical coupling, therefore, represents a critical therapeutic strategy that ensures adequate ROS production for effective biofilm eradication while preserving tissue biocompatibility. The self-powered strategy of the CPU gels can smooth the deficiencies of static antimicrobial agents, such as diminishing efficacy over time while avoiding antibiotic resistance linked to conventional approaches [62]. By harnessing the energy of intermittent motion, the mechano-powered CPU system has achieved a dynamic sustained antibacterial activity without external energy input or chemical overuse.

In future studies, we will systematically evaluate different sterilization methods (autoclaving, gamma irradiation, low-temperature plasma) on material properties to identify the optimal clinical protocol. Concurrently, we will explore various approaches to enhance material stability, including encapsulation under an inert gas atmosphere, to prolong shelf life. Special attention will be given to optimizing setting time for clinical operability. Further validation will include performance testing under simulated chewing loads and comprehensive biocompatibility assessment to ensure clinical applicability.

## 3. Conclusions

A novel mechano-responsive therapeutic approach has been successfully developed through an injectable conductive polyurethane (CPU) composite gel incorporating piezoelectric n-BaTiO_3_ nanoparticles and functional components. Under cyclic compressive loading, the formation of an interconnected piezoelectric/conductive network within the composite matrix enables efficient charge transport via mechano-electric coupling. This design results in enhanced sensitivity to mechanical stimuli, with intermittent motion being converted into sustained charge transport and ROS generation, synergistically combined with chemical antimicrobial activity for improved bacteriostasis and biofilm eradication. The experimental results confirm exceptional biofilm eradication efficiency (97%) alongside maintained biocompatibility, representing a significant advancement from conventional passive materials to actively responsive therapeutic systems for prolonged infection control in root canal therapy.

## 4. Materials and Methods

### 4.1. Materials

The key materials were sourced from commercial suppliers (Shanghai Aladdin (Shanghai, China); Chengdu Kelong (Chengdu, China)): PTMEG (*M*_n_ = 2000), Isophorone Diisocyanate (IPDI), nano-zinc oxide (n-ZnO), nano-bismuth oxide (n-Bi_2_O_3_), nano-barium titanate (n-BaTiO_3_), polyethylene glycol (PEG_600_, *M*_n_ = 600), stannous salt, trolamine, aniline, and p-phenylenediamine. Nano-hydroxyapatite (n-HA) particles were synthesized via chemical precipitation (method detailed previously) followed by vacuum-drying (108 °C, 2 h) [71]. All the chemicals were analytical grade.

### 4.2. Synthesis of AT

On the basis of previous studies, amino-terminated aniline trimers (ATs) were synthesized [72]. In brief, after the p-phenylenediamine was dissolved in a solution, ammonium persulfate was added under circulated cooling. When the color of the solution changed, aniline was added till the reaction ended and the crude products were filtered; washed with hydrochloric acid, distilled water, and ammonia water; and dried by a freeze dryer. AT product was then purified by a rotary evaporator and ground into a powder state with an agate mortar. The purification process removes unreacted monomers and solvents, effectively preventing cytotoxicity or inflammatory reactions caused by these impurities, thereby significantly improving the biocompatibility of the material [35].

### 4.3. Preparation of Injectable Conductive Polyurethane-Based Composite Gels

#### 4.3.1. Component A: Injectable Prepolymer

PTMEG and mannitol were mechanically mixed in a three-neck flask under N_2_ and thermally processed at 70 °C for 1 h. Then, n-HA as a calcified promoter with good biocompatibility, Bi_2_O_3_ as an X-ray inhibitor with radiopacity, and n-ZnO (the hexagonal fibrous zincite structure (Figure S2A) [73]) as a chemical fungicide providing broad-spectrum bacteriostasis were added referring to a previous study [24] and dispersed for 1 h with/without piezoelectric n-BaTiO_3_ addition (Figure S2B) (gels’ composition shown in Table 1) [74]. Stage II comprised the controlled addition of IPDI/AT under a nitrogen atmosphere, followed by pre-polymerization at 75 °C for 2 h, with rigorous maintenance of the isocyanate index at a molar ratio of NCO:OH = 1.5 throughout the process.

#### 4.3.2. Component B: Curing Reagent

The curing reagent, referred to as component B, was prepared by mixing triethanolamine, polyethylene glycol (PEG_600_), and tin salt at a mass ratio of 45:17:1. The mixture was then subjected to ultrasonication for 30 min to ensure thorough homogenization.

#### 4.3.3. Preparation of Gels

Components A and B were combined at a 20:1 volume ratio under ambient temperature, thoroughly mixed to form a homogeneous paste, and subsequently transferred into a cylindrical polytetrafluoroethylene mold (6 mm diameter × 12 mm height). The filled mold was then thermally cured in a controlled-temperature oven maintained at 37 °C. Four groups of polyurethane composite gels were fabricated as preceding steps and the abbreviated names and formulations of each group were shown in Table 1. After the mixtures had been cured in the Teflon mold (Φ 10 mm **×** 2 mm), we removed them from the mold and dried and sterilized them through low-temperature plasma sterilization for electrical and biological tests.

### 4.4. Physicochemical Evaluation of Gels for Root Canal Therapy

#### 4.4.1. XRD and FT-IR

The copolymers had been characterized by the X-ray diffraction of Cu Kα radiation (XRD, EMPYERAN, Panalytical, Almelo, The Netherlands). Scans and 2 θ range was from 10 to 80° at a step size of 0.03° under the conditions of 30 kV and 25 mA. The FTIR spectra (Nicolet 6700, PerkinElmer, Waltham, MA, USA) of PU composites were recorded from 650 to 4000 cm^−1^ with 4 cm^−1^ resolution to analyze molecular interactions.

#### 4.4.2. Anti-Washout Property

After components A and B were mixed evenly, the mixture was immediately transferred to a syringe, injected into a Petri dish, and then injected with 10 mL of deionized water at room temperature. The washout resistance of the composites was evaluated according to the method in previous research [75].

#### 4.4.3. Material Morphology and Element Distribution

Morphological analysis was conducted using a JEOL JSM-7500F scanning electron microscope, with coupled energy-dispersive X-ray spectroscopy (EDS) employed of Ca, Bi, Zn, and Ba across the gel surfaces. Prior to imaging, specimens underwent gold sputter-coating to ensure surface conductivity under high-vacuum conditions.

#### 4.4.4. Setting Time and Flowing

The flowability and setting time of the gels were tested in accordance with ISO 6876: 2012 [38]. After the A and B components were mixed, the mixed slurry was injected into the Teflon mold (Φ 5 mm × 3 mm) and then placed in the oven at 37 °C. Setting time was defined as the duration required for the material to achieve structural resistance against Gillmore needle penetration. Each group of samples was measured three times.

After mixing component A and component B for 180 ± 5 s, we used a syringe with a scale to inject (0.05 ± 0.001) ml of gels into a glass plate center (20 g), and then placed a glass plate (20 g) on top of the gels and place a 100 g weight on it. After 10 min, diameters (max/min) were measured with vernier calipers through triplicate testing per sample group.

#### 4.4.5. Degree of Crosslinking

According to the expansion equilibrium method, the degree of crosslinking was evaluated through the characterization of the swelling rate and gel in the polyurethane composite gels. Following the stoichiometric homogenization of components A/B, the reactive mixture was cast into cylindrical PTFE molds (Φ 6 × 12 mm) and cured in the oven at 37 °C, where the original weight of the cured sample was ***M_1_***, and then the sample was soaked in tetrahydrofuran alcohol for 24 h. After the soaked sample was removed and the liquid on its surface was wiped clean, the wet weight was weighed as ***M_2_***. Finally, the sample was dried in an oven at 50 °C till its weight no longer changed; thus, its dry weight was weighed to ***M_3_***. Each group of samples was measured three times. The swelling rate and gel fraction of polyurethane material were calculated by Formulas (1) and (2).

(1) $$Q=(M_{2}-M_{1})/M_{1}$$

(2) $$G=M_{3}/M_{1}$$

where ***Q*** was the swelling rate of the polyurethane material, ***G*** was the gel fraction of the polyurethane material, ***M_1_*** was the original weight of the polyurethane material, ***M_2_*** was the mass of the polyurethane material after soaking in tetrahydrofuran for 24 h, and ***M_3_*** was the mass of the polyurethane material after drying.

#### 4.4.6. Mechanical Test

Test specimens were fabricated following Section 4.3.3, injected into Teflon molds (Φ 6 mm × 12 mm), and oven-cured at 37 °C. Mechanical characterization utilized an AUTOGRAPH AG-IC 20/50KN tester (Tokyo, Japan) at 1 mm/min displacement until the 50% compression of initial length (25 °C). Compression modulus was determined from linear stress–strain curve slopes. Each group tested five independent samples.

### 4.5. Electrochemical Properties of Composite Gels Under Compressive Loading

#### 4.5.1. Conductivity and Electrochemical Properties

The electrical conductivity of the cured specimens was measured using a Keithley 6517A 4-probe tester equipped with 1.0 mm-spaced linear probes. Each set of experiments tested three samples. The conductivity was calculated by Formula (3).

(3) δ=1/ρ

where ***δ*** represented conductivity and ***ρ*** represented the resistance.

The cured samples’ electroactivity was analyzed via UV-vis spectroscopy (PerkinElmer Lambda 35) using DMF solution. Spectral acquisition employed the same spectrophotometer configuration.

#### 4.5.2. Piezoelectric Properties Under Cyclic Compressive Loading

A piezoelectric impact test was conducted at 1.0 Hz using a linear motor as the impact source. The voltage output from the PEH devices was measured using a Keithley 6514 electrometer, while the current output was recorded with a low-noise current preamplifier (SR570, Stanford Research Systems, Sunnyvale, CA, USA). A DAQ card (NIPCI-6221, Beijing Altai Technology Company, Beijing, China) acquired and processed the signals. The ability and types of ROS generation of the mechano-powered gels were tested by the colorimetric method of 3,3′,5,5′-tetra- methylbenzidine (TMB) and 1,3-diphenyl isobenzofuran (DPBF) to screen suitable parameters of cyclic compressive loading. The screening process is detailed in Figure S1 (Supplementary Materials). The optimized compressive loading was as follows: 10 N for 30 cycles (half a minute per cycle). The cured samples were divided into two groups: one group was subjected to an axial compressive load of 10 N for 30 cycles, and the other group was not subjected to the compressive load. Each group of samples was measured three times. Under the same loading conditions, ZJ-3A was used to test the piezoelectric constant of the cured material.

### 4.6. Antibacterial Properties Under Cyclic Compressive Loading

The cured samples were split into two groups. One group was applied a 10 N force for 30 cycles (half a minute per cycle) and the other group was not under compression; thereafter, the same mechanical conditions were loaded in the following biological tests.

Gram-negative *Escherichia coli* (*E. coli*, ATCC 25922) and Gram-positive *Staphylococcus aureus* (*S. aureus*, ATCC 25923) evaluated the antibacterial properties of the conductive polyurethanes gels in the absence of load and under cyclic compressive load. *E. coli* and *S. aureus* were grown in Oxoid broth at 37 °C with oxygen. After centrifugation, the bacteria were transferred to fresh broth. Bacterial suspensions were adjusted to specific optical densities matching known concentrations to prepare inocula. The antimicrobial activity of the polyurethane gels was evaluated using two methods: static exposure conditions following ASTM G21-15 and dynamic exposure conditions based on ASTM E2149-2013a.

#### 4.6.1. Static Contact Test (SCT)

After curing in a Φ 10 mm × 2 mm Teflon mold, the mixtures were removed and ground. After curing, the samples were immersed in 10 mL PBS and oscillated in a 37 °C water bath for 0, 3, 7, and 15 days. All the samples underwent low-temperature plasma sterilization before testing. When the cured sample was placed in the center of the Petri dish, 20 μL of suspended bacterial solution (2 × 10^6^ CFU per mL) was added to the surface of the sample. The sterilized samples were incubated in a humid atmosphere at 37 °C for 2 h to evaporate the bacterial suspension under different loading conditions to ensure direct contact between the bacteria and the sample. Next, the specimen surfaces were washed three times with 5 mL of phosphate-buffered saline (PBS), and the wash solutions were collected. A 1 mL aliquot of the collected wash liquid was diluted in 9 mL PBS, after which 200 μL of this diluted sample was plated onto Brain Heart Infusion (BHI) agar. The resulting colonies were enumerated (***C_2_***) following 48 h of incubation. A sterile, dry polyethylene film (10 mm diameter) served as the negative control (***C_1_***). The bacteriostatic rate was calculated using Equation (4), and the experiment was replicated three times.

(4) $$Bacteriostatic\;rate\;(\%)=(C_{1}-C_{2})/C_{1}\times 100\%$$

#### 4.6.2. Dynamic Contact Test (DCT)

The fresh mixture of different composites was coated on the bottom and side walls of the 96-well plate. The material was immersed in PBS solution for 15 days. Following the extraction of the immersed solution, all the coated 96-well plates were sterilized using low-temperature plasma sterilization. Then, 20 μL of bacterial suspension ((3 − 7) × 10^6^ CFU per mL) was added dropwise to the coated well and cultured under different loading conditions for 2 h at 37 °C: one group was subjected to a cyclic 10 N force (30 cycles, with each cycle lasting 30 s), while the other group remained uncompressed. In the process, the bacterial suspension was evaporated, ensuring that the bacteria were in direct contact with the curing gel surface. Next, 200 μL of culture solution was added to each well. Absorbance readings were taken at 0, 1, 2, 3, 4, 5, 6, 7, 8, and 24 h post-incubation using a microplate reader (PerkinElmer 1420 Multilabel Counter, PerkinElmer, Waltham, MA, USA) at 650 nm. Sterilized, uncoated wells served as the blank control group. Three replicates were measured for each treatment group.

### 4.7. In Vitro Bacterial Biofilm Culture and Eradication Under Cyclic Compressive Loading

*Escherichia coli* (*E. coli*, ATCC 25922) and *Staphylococcus aureus* (*S. aureus*, ATCC 25923) strains were cultured in broth medium (Oxoid Ltd., Basingstoke, UK) at 37 °C. Next, 1 × 10^8^ CFU/mL of bacteria were inoculated into 12-well plates and incubated at 37 °C for 48 h to form bacterial biofilms. The unbound bacteria were rinsed with PBS and then the sterilized samples were placed tightly on the biofilm under different loading conditions for 2 h. To quantify residual biofilm biomass, the biofilms were stained with 0.1% crystal violet for 30 min. The stained biofilm was washed with PBS and dispersed in 1.0 mL of 95% ethanol, and the remaining biofilm biomass was quantified by measuring absorbance at 590 nm. The relative biofilm mass was calculated by Formula (5).

(5) $$Relative\;biofilm\;mass\;\%=A_{1}/A_{2}\times 100\%$$

where ***A_1_*** and ***A_2_*** represented the absorbance values of crystal violet solution at 590 nm for the experimental group and control group, respectively.

Bacterial viability was assessed via plate counting after disrupting the treated biofilms. For the morphological analysis, the biofilms were fixed with 2.5% glutaraldehyde (2 h) and examined using a JSM-7500F SEM (Jeol, Tokyo, Japan).

### 4.8. Chemical Structural Changes and ROS Generation Under Cyclic Compressive Loading

In order to test the piezoelectric effect of conductive polyurethane gels under cyclic compressive loading on AT segments of molecular chain, the cured samples (1 cm × 1 cm × 5 mm) were divided into two groups. One group was subjected to 10 N loading for 30 cycles (half a minute per cycle), and the other group was not under compression. The material’s chemical structure was analyzed using X-ray photoelectron spectroscopy (XPS, Thermo Scientific K-Alpha, USA).

Considering biofilm is affected by the reactive oxygen species (ROS), the colorimetric method of TMB and DPBF was used to investigate the ability and types of ROS generation of cured gels under cyclic loading. The generation of •O_2_^−^ was verified using a Nitrotetrazolium Bluechloride (NBT) assay [46]. It was dispersed in sodium citrate buffer (pH 5.0) with TMB as substrate.

The cured samples were placed in the TMB solution under the compression of 10 N loading for 30 cycles (half a minute per cycle), respectively, wherein the samples were soaked in the solution for 30 min without load as a contrast. Then, the UV-vis spectroscopy of the solution was measured by UV-vis spectrophotometer. The material was placed in PBS solution and the corresponding amount of NBT developing agent was added. The same treatment was applied to the material, and the UV-vis spectrum of the solution was determined by UV-vis spectrophotometer. Finally, the DPBF was dispersed in PBS (pH 7.4). The cured samples were placed in the DPBF-PBS solution loading with the pressure of 10 N for 30 cycles (half a minute per cycle), respectively, wherein the samples were soaked in the solution for 30 min without pressure as a contrast. Then, the UV-vis spectroscopy of the solution was measured by UV-vis spectrophotometer.

### 4.9. In Vitro Cell Evaluation Under Cyclic Compressive Loading

#### 4.9.1. Cell Culture and Extraction Solution Preparation

The L929 mouse fibroblast cell line was provided by West China School of Stomatology, Sichuan University. The L929 cells were cultured in DMEM medium (Gibco, USA) with 10% fetal bovine serum (FBS, Gibco) at 37 °C in a 5% CO_2_ atmosphere. The cells were subcultured at 80% confluence, and the medium was refreshed every other day.

The preparation of the extracted solution of the different gels was according to ISO 10993-9 [76]. The sterilized samples were immersed in DMEM (Gibcos; Thermo Fisher Scientific, Waltham, MA, USA) medium supplemented with 10% fetal calf serum (FBS, Gibco). The samples were immersed at 0.1 g/mL and stored in a 5% CO_2_ atmosphere (37 °C, 24 h). The extract was collected and stored at 4 °C.

#### 4.9.2. Cell Proliferation

Cell proliferation was assayed by Cell Counting Kit-8 (CCK-8, KeyGEN BioTech, Nanjing, China). The L929 cells were seeded in 24-well plates (2 × 10^4^ cells/well) and cultured for 1, 4, and 7 days in a 5% CO_2_ atmosphere at 37 °C. The culture medium was replaced every 2 d. The cured material was trimmed into 1 cm × 1 cm × 5 mm squares, which were completely wrapped with conductive glue. Two conductive wires were drawn from the upper and lower sides. The orifice plates were divided into two groups, one of which did not apply additional treatment. The other group inserted conductive wires into the orifice plates and dipped them into the medium. The sterilized samples were then subjected to pressure under different loading conditions. Each well received 900 μL fresh medium and 100 μL CCK-8 solution, followed by 2 h incubation at 37 °C. The solution was transferred to a 96-well plate, and absorbance at 450 nm was measured using a PerkinElmer Wallac Victor 31420 microplate reader. Triplicate samples were tested per group.

#### 4.9.3. Live-Dead Cell Staining Assay

The L929 cells were seeded in 24-well plates (2 × 10^4^ cells/well) and cultured for 1, 4, and 7 days in a 5% CO_2_ atmosphere at 37 °C. The cured material was trimmed into 1 cm × 1 cm × 5 mm squares, which were completely wrapped with conductive glue. Two conductive wires were drawn from the upper and lower sides. The orifice plates were divided into two groups, one of which did not apply additional treatment. The other group inserted conductive wires into the orifice plates and dipped them into the medium. The sterilized samples were then subjected to pressure under different loading conditions. Cell viability was assessed using a LIVE/DEAD stain (Invitrogen, Waltham, MA, USA) following the manufacturer’s protocol. The stained cells were visualized with a Nikon fluorescence microscope (Tokyo, Japan).

### 4.10. Statistical Analysis

The experimental data are presented as mean ± standard deviation (SD). Statistical evaluations were performed using the SPSS software (version 25.0; LEAD Technologies, Chicago, IL, USA) through one-way ANOVA followed by Tukey’s or Dunnett’s post hoc tests. Significance thresholds were defined at *p* < 0.05 (statistically significant), *p* < 0.01 (highly significant), and *p* < 0.001 (exceptionally significant), with *p* > 0.05 indicating non-significant differences.

## Figures and Tables

**Table 1:** Composition and abbreviation of the injectable and conductive polyurethane gels.

	I	IC	IC-B1	IC-B2
AT (wt%)	0	2.5	2.5	2.5
BaTiO_3_ (wt%)	0	0	5	10

Abbreviations of the composite gels: **I**: injectable polyurethane composite (polyurethane/n-HA/Bi_2_O_3_/n-ZnO at a weight ratio of 13: 2: 4: 1); **IC**: injectable conductive polyurethane composite; **IC-B**: injectable conductive polyurethane composite with the addition of n-BaTiO_3_.

## Data Availability

All the data supporting the conclusions are presented in the manuscript and the Supplementary Materials.

## Supplementary Materials

The following supporting information can be downloaded at: https://www.mdpi.com/article/10.3390/gels11050346/s1, Figure S1: ROS generation of gels under 10 N compression loading for 10, 20 and 30 cycles. (A) IC-B1 and (B) IC-B2: The production of ·OH was detected by TMB. (C) Peak value of IC-B1 and IC-B2 at 652 nm under cyclical loading for 30 cycles. (D) IC-B1 and (E) IC-B2: The production of ·O_2_- was detected by NBT. (F) Peak value of IC-B1 and IC-B2 at 560 nm under cyclical loading for 30 cycles. (G) IC-B1 and (H) IC-B2: The production of 1O_2_/·O_2_- was detected by DPBF. (I) Peak value of IC-B1 and IC-B2 at 420 nm under cyclical loading for 30 cycles; Figure S2: XRD patterns of (A) n-ZnO and (B) n-BaTiO_3_. (C) Magnified XRD pattern of n-BaTiO_3_; Figure S3: FT-IR spectra of the AT; Figure S4: Properties of different gels in curing process. (A) The representative FTIR spectra of gel with cure time (2 h and 12 h). (B) Isocyanate conversion calculated by the integrated peak areas of representative FTIR spectra (at 2200–2300 cm^−1^) during curing of copolymers (2 h). (C) Diagram of temperature variation during curing of copolymers; Figure S5: SEM images of polyurethane gels after cured (×2000) and element distribution of Ca, Bi, Zn, and Ba on the surface of cured samples; Figure S6: Anti-washout property of gels after setting; Figure S7: Antibacterial properties of cured seals in the absence of loading. Antibacterial rates of static contact experiment against (A) *S. aureus* and (B) *E. coli*. Dynamic contact test curves of the various groups after soaking in PBS solution for 15 d against (C) *S. aureus* and (D) *E. coli*. The uncoated wells were set up as the blank control group; Figure S8: Proliferation of L929 cells in polyurethane gels under 10 N compression loading for 30 cycles or unloaded condition. (A) Schematic diagram of experimental apparatus. (B) The OD values of CCK-8 were determined after L929 cells were cultured in polyurethane gels extract for 1, 4, and 7 days (one group was connected to the culture medium through a wire when pressure was applied to the solid material, and the other group was not subjected to other operations). * *p* < 0.05, ** *p* < 0.01, *** *p* < 0.001. (n = 3); Table S1: Gel fraction of polyurethane gels; Table S2: Average molecular weight and polydispersion coefficient of p-I and p-IC prepolymers; Table S3: Comparison of Antibacterial Rates and Biofilm Inhibition Efficiencies Against *E. coli* and *S. aureus* Among Selected Commercial Materials, Literature-Reported Materials, and IC-B2 Gel; Video S1: Video demonstration of the injectable property of the gels.
