# Supplementary material for: Injectable and Conductive Polyurethane Gel with Load-Responsive Antibiosis for Sustained Root Canal Disinfection
Source: Gels. 2025 May 7;11(5):346. doi: 10.3390/gels11050346 (PMC12111077; doi:10.3390/gels11050346)
Supplement: Supplementary file 1 [file gels-11-00346-s001.zip › Supplementary Information.pdf]

Supporting Information

Injectable and conductive polyurethane gel with load-responsive antibiosis for sustained root canal disinfection

Bo Mu[a], Xiaoyu Lei[a], Yinglong Zhang[a], Jingzheng Zhang[a], Qingda Du[a], Yuping Li[a], Dongyu Huang[a], Li Wang[b], Jidong Li[a], Yubao Li[a], Yi Zuo[a,*]

[a]Research Center for Nano Biomaterials, Analytical & Testing Center, Sichuan University, Chengdu 610064, PR China

[b]School of Big Health and Intelligent Engineering, Chengdu Medical College, Chengdu 610500, PR China

[*]Corresponding author. Email address: zoae@scu.edu.cn (Y. Zuo)

Materials and methods

Optimization of cyclic compression load parameters:

The size of the cyclic compression load: According to clinical statistics, the average chewing force of human premolars is 10.5 ± 1.7 N [S1]. Therefore, 1 kg weight as a pressure source will be applied in next testing to mimic the average force of human mouth teeth during occlusion.

Cycle number of cycles of compression load: Considering the effect of reactive oxygen species (ROS) on biofilm, the number of cycles was determined by the efficiency of ROS generation in conductive polyurethane gels under cyclic compression load. The ability of curing sealant to produce reactive oxygen species under cyclic pressure was studied by TMB and DPBF colorimetry. The formation of $\bullet O_2^-$ was determined using NBT [S2]. The cured samples were placed in the TMB solution under the compression of 10 N loading for 10, 20, 30 cycles (half a minute per cycle)

respectively. The material was placed in PBS solution and the corresponding amount of NBT developing agent was added. The same treatment was applied to the material. Finally, the DPBF was dispersed in PBS (pH 7.4). The cured samples were placed in the DPBF-PBS solution loading with the pressure of 10 N for 10, 20, 30 cycles (half a minute per cycle) respectively. Then the UV-vis spectroscopy of the solution was measured by UV-vis spectrophotometer.

^{1}H NMR: The ^{1}H NMR (400 MHz) spectra of AT was obtained by a Bruker Ascend 400 MHz NMR instrument with the solution of DMSO-d6. This test was carried out at room temperature and DMSO-d6 as internal standard (δ 2.50 ppm).

Gel permeation chromatography: GPC measurements were conducted at 35 °C by using the Alliance e2695 (GPC, Waters, America). Tetrahydrofuran (THF) was chosen as eluent and the flow rate was 0.6 mL/min to determine the average molecular weight and polydispersity index (PDI) of Component A prepolymer. Polystyrene standards (Shodex SM-105) were used as calibration of molecular weight standard curve.

Heat release in curing process: After the mixture of component A and component B is evenly mixed according to the volume ratio, 3 ml of the mixture is injected into the polypropylene mold (Φ 6 mm × 12 mm) and placed in the oven at 37 °C. The temperature was measured at different curing time points (10 min, 20 min, 30 min, 60 min, 90 min, 120 min).

Isocyanate conversion during curing: After component A and component B were mixed evenly according to the volume ratio, the total reflection was measured by Fourier infrared spectrometer at different curing time points (10 min, 20 min, 30 min, 40 min, 60 min, 90 min, 120 min, 3 h, 4 h, 6 h, 8 h, 10 h, 12 h).

Results

In Figure S1, under the action of cyclic pressure load, the IC-B1 and IC-B2 gels generated different contents of ROS, including of •OH (TMB), $•O_2^-$ (NBT) and $^1O_2$ (DPBF) measured by UV-vis spectrophotometer, respectively. The strength of the generated •OH increased with the increase of cyclic loading period, and the strength of the generated •OH reached the highest after the compressive stress loading of the IC-B2 gel for 30 cyclic cycles (Figure S1A and S1B). The generation of $•O_2^-$ and $^1O_2$ was similar to that of •OH, and had a positive correlation with the cycle numbers of cyclical loading. Increasing the ROS level could continuously and efficiently destroyed bacteria (cell membranes, poly-polysaccharides, proteases, nucleic acids, etc.) and biofilm, thus leading to loss of the ability of metabolism, proliferation, and film formation [S3]. In order to obtain better antibacterial effect of the gel, we chose to apply a cyclic compressive stress of 10 N to the cured gels for 30 cycles in the following experiments (half a minute per cycle).

Figure S2A showed the XRD pattern of n-ZnO, the diffraction peaks at 2θ values of 31.8°, 34.4°, 36.3°, 47.6°, 56.7°, 62.8°, and 68.0° were correspond to the planes (100), (002), (101), (102), (110), (103), and (112), respectively. The n-ZnO is a hexagonal fibrous zincite structure with good crystallinity [S4]. Figure S2B showed the XRD pattern of n-$BaTiO_3$, the diffraction peaks at 2θ values of 22.3°, 31.6°, 38.9°, 50.9°, 56.1°, 65.8°, and 74.8° were correspond to the planes (100), (110), (111), (210), (211), (220), and (310), respectively. Moreover, in the enlarged XRD pattern of n-$BaTiO_3$ in Figure S2C, the peaks corresponding to (002) and (200) are split at 45.1° and 45.5°, indicating that n-$BaTiO_3$ had a tetragonal structure with piezoelectric characteristics [S5].

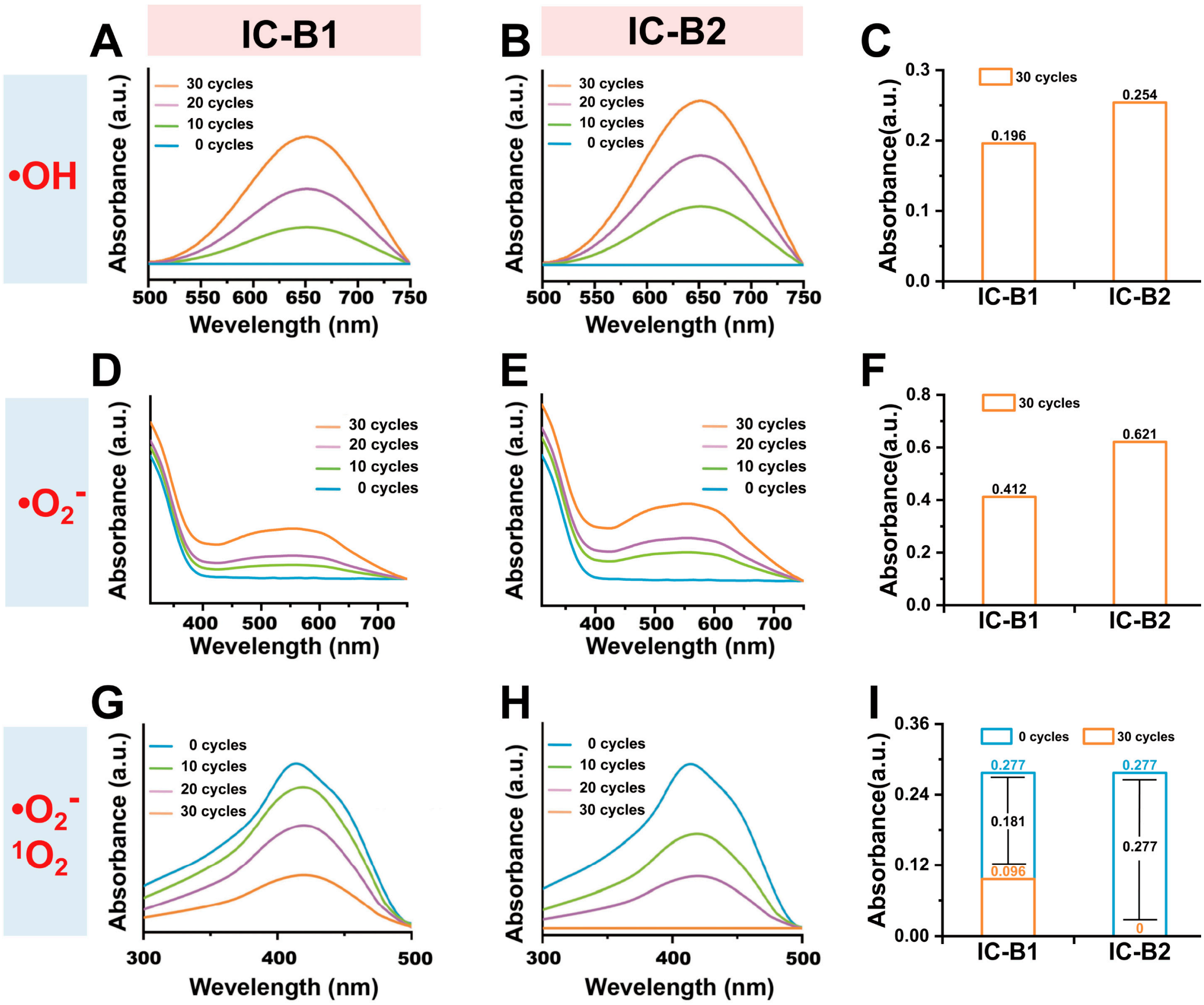

Figure S1. ROS generation of gels under 10 N compression loading for 10, 20 and 30 cycles. (A) IC-B1 and **(B)** IC-B2: The production of ·OH was detected by TMB. **(C)** Peak value of IC-B1 and IC-B2 at 652 nm under cyclical loading for 30 cycles. **(D)** IC-B1 and **(E)** IC-B2: The production of $\cdot O_2^-$ was detected by NBT. **(F)** Peak value of IC-B1 and IC-B2 at 560 nm under cyclical loading for 30 cycles. **(G)** IC-B1 and **(H)** IC-B2: The production of $^1O_2/\cdot O_2^-$ was detected by DPBF. **(I)** Peak value of IC-B1 and IC-B2 at 420 nm under cyclical loading for 30 cycles.

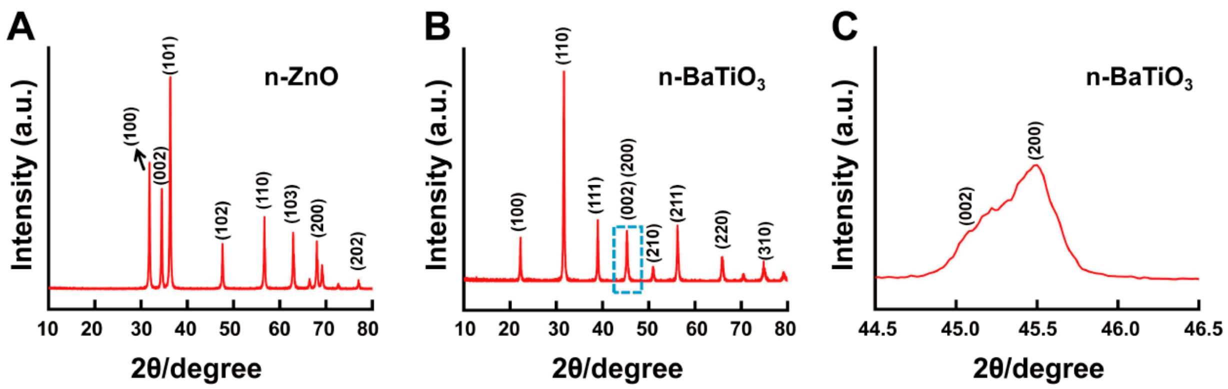

Figure S2. XRD patterns of **(A)** n-ZnO and **(B)** n-$BaTiO_3$. **(C)** Magnified XRD pattern of n-$BaTiO_3$.

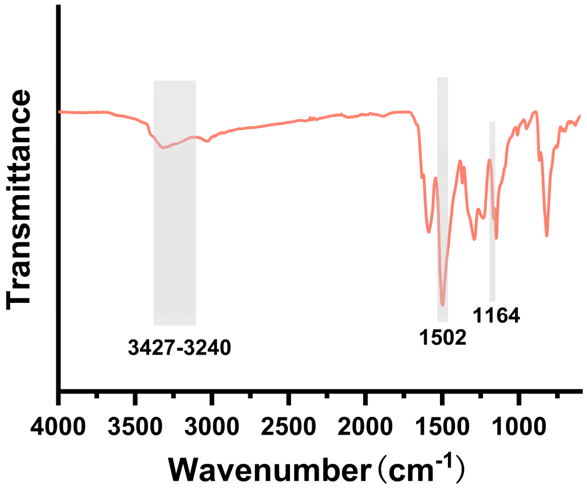

Figure S3. FT-IR spectra of the AT

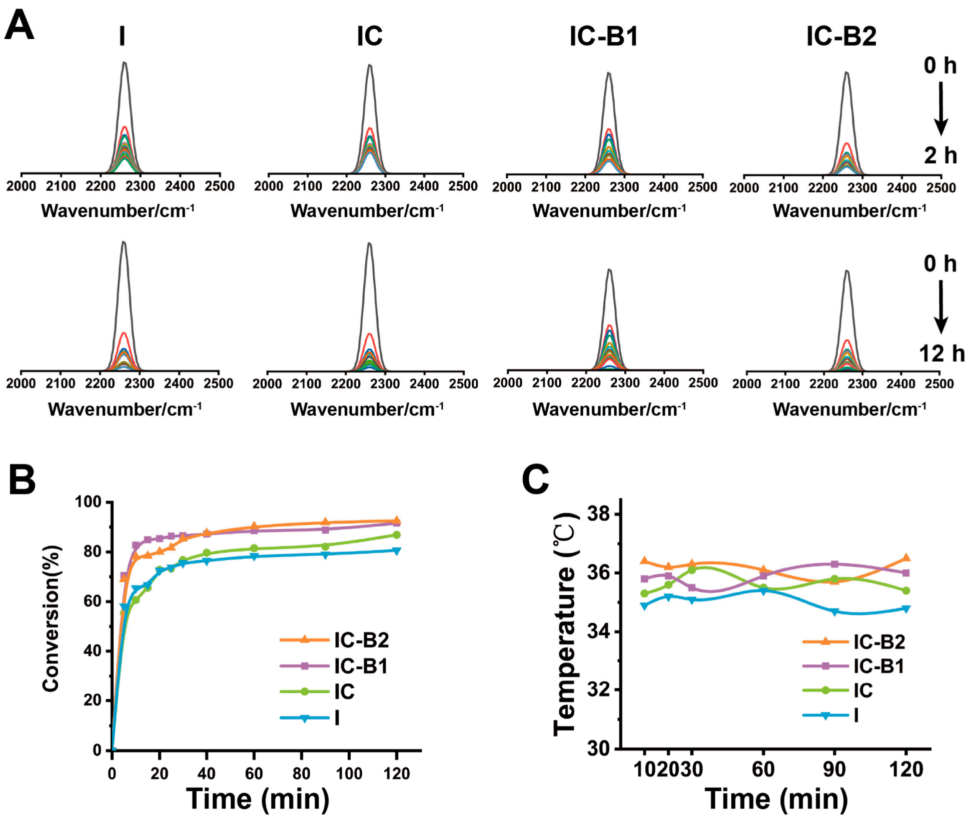

Figure S4. Properties of different gels in curing process. (A) The representative FTIR spectra of gel with cure time (2 h and 12 h). **(B)** Isocyanate conversion calculated by the integrated peak areas of representative FTIR spectra (at 2200-2300 cm^{-1}) during curing of copolymers (2 h). **(C)** Diagram of temperature variation during curing of copolymers.

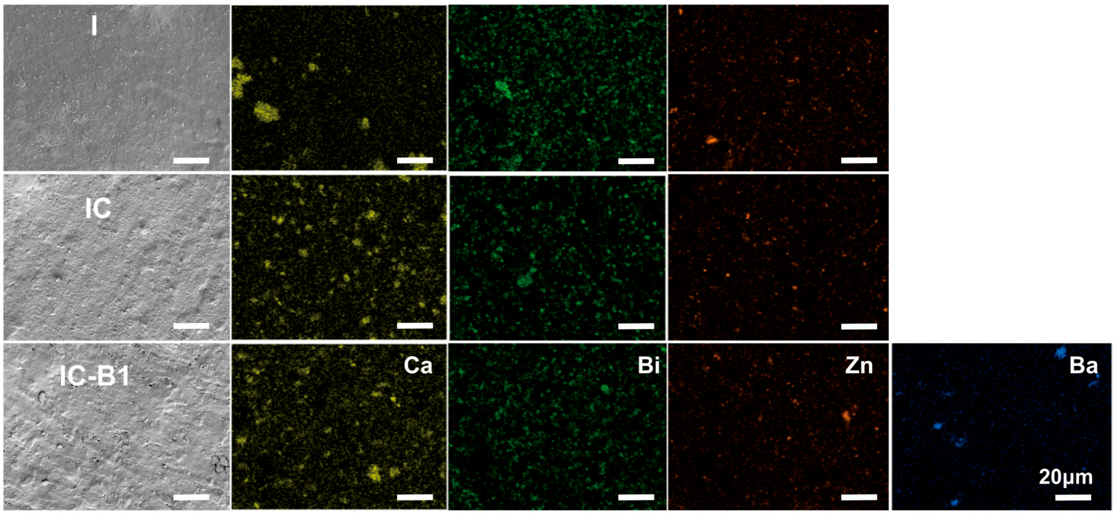

Figure S5. SEM images of polyurethane gels after cured (× 2000) and element distribution of Ca, Bi, Zn, and Ba on the surface of cured samples.

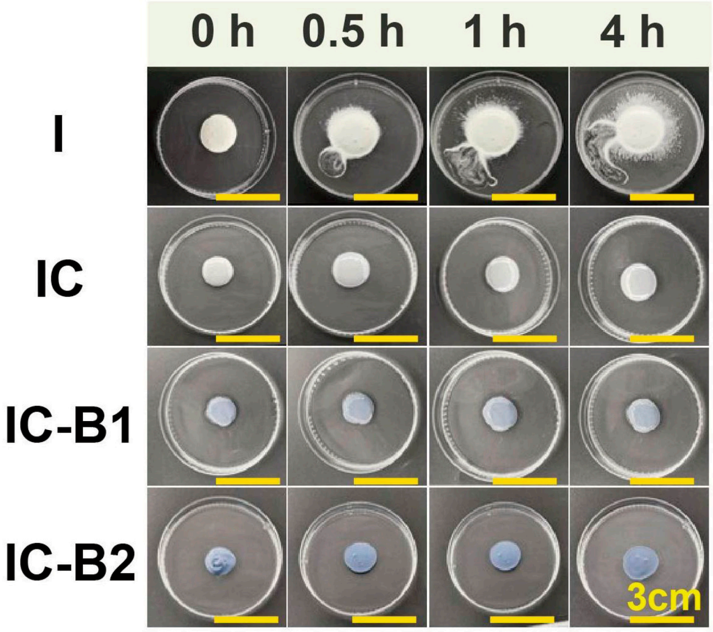

Figure S6. Anti-washout property of gels after setting.

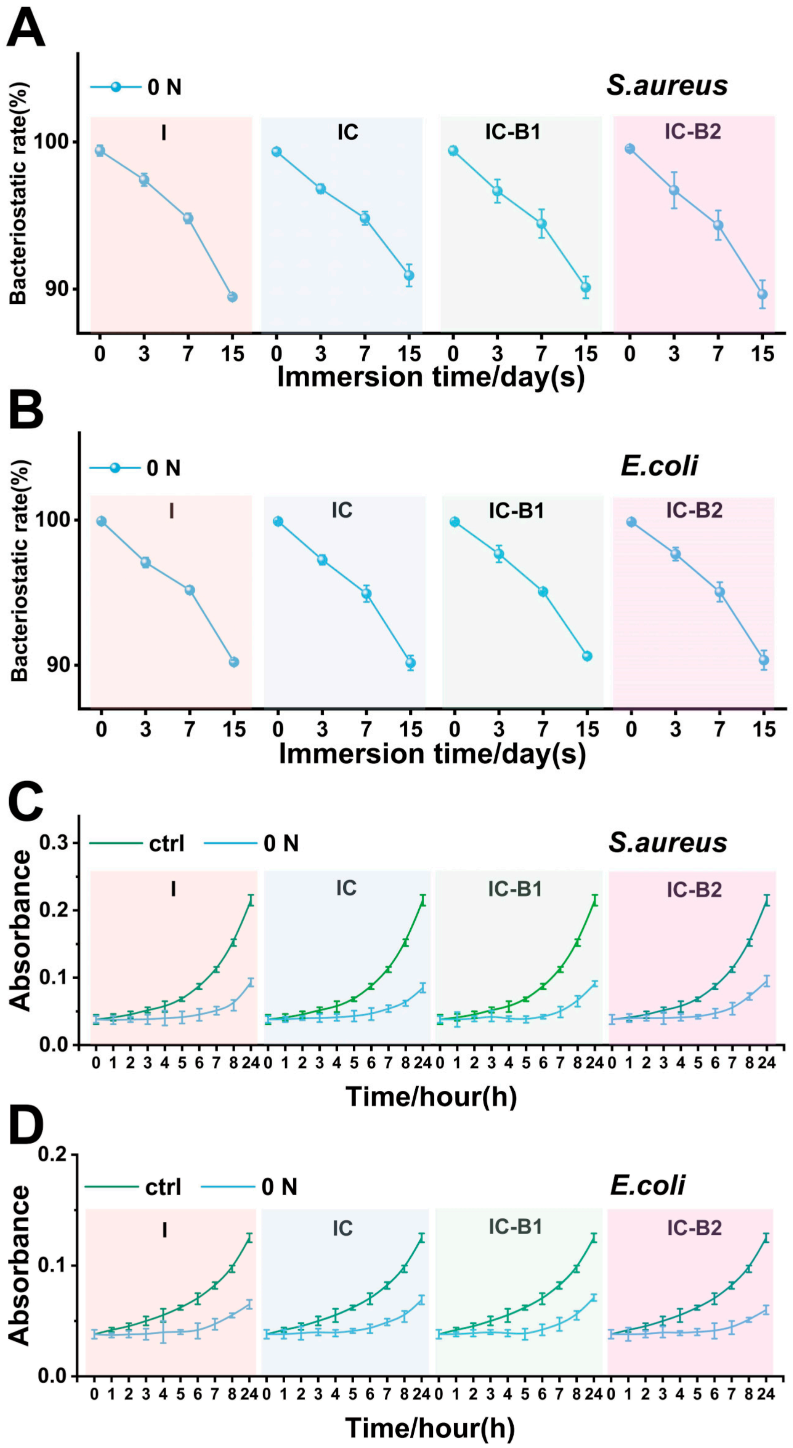

Figure S7. Antibacterial properties of cured seals in the absence of loading. Antibacterial rates of static contact experiment against **(A)** *S. aureus* and **(B)** *E. coli*. Dynamic contact test curves of the various groups after soaking in PBS solution for 15 d against **(C)** *S. aureus* and **(D)** *E. coli*. The uncoated wells were set up as the blank control group.

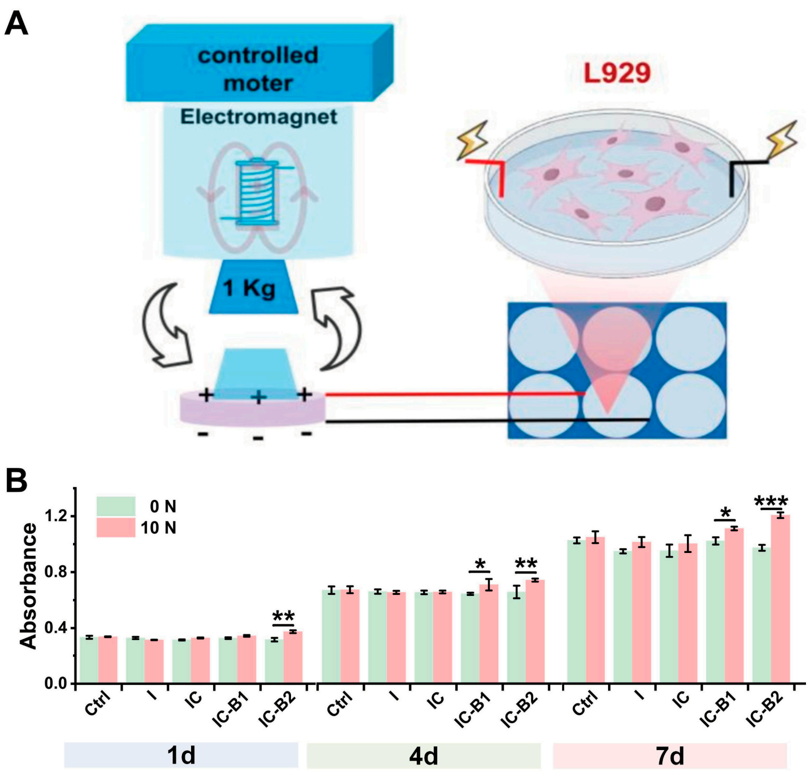

Figure S8. Proliferation of L929 cells in polyurethane gels under 10 N compression loading for 30 cycles or unloaded condition. (A) Schematic diagram of experimental apparatus. **(B)** The OD values of CCK - 8 were determined after L929 cells were cultured in polyurethane gels extract for 1, 4, and 7 days (one group was connected to the culture medium through a wire when pressure was applied to the solid material, and the other group was not subjected to other operations). * $p < 0.05$, ** $p < 0.01$, *** $p < 0.001$. (n= 3).

Table S1 Gel fraction of polyurethane gels

Sample	I	IC	IC-B1	IC-B2
Gel fraction (%)	98.34 ± 0.29	98.64 ± 0.29	98.92 ± 0.47$^{(*)}$	98.68 ± 0.19$^{(*)}$

(n = 3) * $p < 0.05$ versus I group.

Table S2 Average molecular weight and polydispersion coefficient of p-I and p-IC prepolymers

Sample	p-I	p-IC	p-IC-B1	p-IC-B2
M_n (kg/mol)	5.830	6.672	9.299	14.797
PDI	2.77	2.14	2.06	2.30

Table S3 Comparison of Antibacterial Rates and Biofilm Inhibition Efficiencies Against *E. coli* and *S. aureus* Among Selected Commercial Materials, Literature-Reported Materials, and IC-B2 Gel

Sample	Antibacterial Effect	Biofilm Resistance Effect	Test Conditions
Epiphany SE [S6, 7]	Antibacterial effect lasts <1 week	No biofilm resistance effect	Static conditions
Pulp Canal Filling EWT [S8]	Antibacterial effect lasts <1 week	Not explicitly mentioned	Static conditions
EndoSequence BC [S8]	Antibacterial effect lasts <1 week	Not explicitly mentioned	Static conditions
AH Plus® / Apexit® Plus [S9]	Antibacterial rate drops to ~30% after 15 days	No biofilm resistance effect	Static conditions
Shah's $BaTiO_3$ Nanoparticles [S10]	Not quantified	85 ± 3.5% biofilm resistance rate	Static conditions (against *S. aureus* only)

Sample	Antibacterial Effect	Biofilm Resistance Effect	Test Conditions
IC-B2	~95% antibacterial rate after 15 days	~97% biofilm resistance rate	Cyclic loading (10 N, 30 cycles)

References

S1 Li J, Zhao X, Xia Y, Qi X, Jiang C, Xiao Y, Jiang F, Jiang X, Yuan G. Strontium-Containing Piezoelectric Biofilm Promotes Dentin Tissue Regeneration. Advanced Materials 2024;36.

S2 Yang L, Tian B, Xie Y, Dong S, Yang M, Gai S, Lin J. Oxygen-Vacancy-Rich Piezoelectric BiO_{2-x} Nanosheets for Augmented Piezocatalytic, Sonothermal, and Enzymatic Therapies. Advanced Materials 2023;35.

S3 Dai C, Lin J, Li H, Shen Z, Wang Y, Velkov T, Shen J. The Natural Product Curcumin as an Antibacterial Agent: Current Achievements and Problems. Antioxidants 2022;11.

S4 Ning X, Hao A, Cao Y, Hu J, Xie J, Jia D. Effective promoting piezocatalytic property of zinc oxide for degradation of organic pollutants and insight into piezocatalytic mechanism. Journal of Colloid and Interface Science 2020;577:290-9.

S5 Zhang W, Feng Q, Hosono E, Asakura D, Miyawaki J, Harada Y. Tetragonal Distortion of a $BaTiO_3/Bi_{0.5}Na_{0.5}TiO_3$ Nanocomposite Responsible for Anomalous Piezoelectric and Ferroelectric Behaviors. ACS Omega 2020;5:22800-7.

S6 Faria-Junior NB, Tanomaru-Filho M, Berbert FL, Guerreiro-Tanomaru JM. Antibiofilm activity, pH and solubility of endodontic sealers. Int Endod J 2013;46:755-62.

S7 Zhang H, Shen Y, Ruse ND, Haapasalo M. Antibacterial activity of endodontic sealers by modified direct contact test against Enterococcus faecalis. J Endod 2009;35:1051-5.

S8 Baras BH, Melo MAS, Thumbigere-Math V, Tay FR, Fouad AF, Oates TW, Weir MD, Cheng L, Xu HHK. Novel Bioactive and Therapeutic Root Canal Sealers with Antibacterial and Remineralization Properties. Materials 2020;13.

S9 Lei X, Wang J, Chen J, Gao J, Zhang J, Zhao Q, Tang J, Fang W, Li J, Li Y, Zuo Y. The in vitro evaluation of antibacterial efficacy optimized with cellular apoptosis on multi-functional polyurethane sealers for the root canal treatment. Journal of Materials Chemistry B 2021;9:1370-83.

S10 Shah AA, Khan A, Dwivedi S, Musarrat J, Azam A. Antibacterial and Antibiofilm Activity of Barium Titanate Nanoparticles. Materials Letters 2018;229:130-3.
